# Revised Temperament and Character Inventory factors predict neuropsychiatric symptoms and aging-related cognitive decline across 25 years

**DOI:** 10.3389/fnagi.2024.1335336

**Published:** 2024-02-21

**Authors:** Lucas Ronat, Michael Rönnlund, Rolf Adolfsson, Alexandru Hanganu, Sara Pudas

**Affiliations:** ^1^Centre de Recherche de l’Institut Universitaire de Gériatrie de Montréal, Neuroimaging of Emotions Lab, Montreal, QC, Canada; ^2^Department of Medicine, Faculty of Medicine, University of Montreal, Montreal, QC, Canada; ^3^Department of Medical and Translational Biology, Umeå University, Umeå, Sweden; ^4^Umeå Center for Functional Brain Imaging, Umeå University, Umeå, Sweden; ^5^Department of Psychology, Umeå University, Umeå, Sweden; ^6^Department of Clinical Sciences, Umeå University, Umeå, Sweden; ^7^Department of Psychology, Faculty of Arts and Sciences, University of Montreal, Montreal, QC, Canada

**Keywords:** personality, cognitive decline, neuropsychiatric symptoms, Alzheimer’s dementia, MRI, longitudinal study

## Abstract

**Introduction:**

Personality traits and neuropsychiatric symptoms such as neuroticism and depression share genetic overlap and have both been identified as risks factors for development of aging-related neurocognitive decline and Alzheimer’s disease (AD). This study aimed to examine revised personality factors derived from the Temperament and Character Inventory, previously shown to be associated with psychiatric disorders, as predictors of neuropsychiatric, cognitive, and brain trajectories of participants from a population-based aging study.

**Methods:**

Mixed-effect linear regression analyses were conducted on data for the full sample (*N*_max_ = 1,286), and a healthy subsample not converting to AD-dementia during 25-year follow-up (*N*_max_ = 1,145), complemented with Cox proportional regression models to determine risk factors for conversion to clinical AD.

**Results:**

Two personality factors, *Closeness to Experience* (CE: avoidance of new stimuli, high anxiety, pessimistic anticipation, low reward seeking) and *Tendence to Liabilities* (TL: inability to change, low autonomy, unaware of the value of their existence) were associated with higher levels of depressive symptoms, stress (CE), sleep disturbance (TL), as well as greater decline in memory, vocabulary and verbal fluency in the full sample. Higher CE was additionally associated with greater memory decline across 25 years in the healthy subsample, and faster right hippocampal volume reduction across 8 years in a neuroimaging subsample (*N* = 216). Most, but not all, personality-cognition associations persisted after controlling for diabetes, hypertension and cardiovascular disease. Concerning risks for conversion to AD, higher age, and *APOE*-ε4, but none of the personality measures, were significant predictors.

**Conclusion:**

The results indicate that personality traits associated with psychiatric symptoms predict accelerated age-related neurocognitive declines even in the absence of neurodegenerative disease. The attenuation of some personality effects on cognition after adjustment for health indicators suggests that those effects may be partly mediated by somatic health. Taken together, the results further emphasize the importance of personality traits in neurocognitive aging and underscore the need for an integrative (biopsychosocial) perspective of normal and pathological age-related cognitive decline.

## 1 Introduction

Two major causes of late-life disability are neurodegenerative impairments and cognitive decline. In turn, Alzheimer’s disease (AD) is considered a major cause of these impairments. AD has been the subject of numerous studies that increased the understanding of the pathophysiological processes, as well as the underlying biological and psychological contributors to this disorder. Several studies have demonstrated that certain personality traits are associated with risk of developing cognitive disorders such as AD. According to two meta-analyses (Low et al., 2013; Terracciano et al., 2014), neuroticism, a tendency toward anxiety, depression, self-doubt and other forms of negative affectivity, increases the risk of AD and mild cognitive impairment. By contrast, conscientiousness, i.e., a tendency to control impulses and engage in goal-directed behaviors, as well as openness to experience, i.e., the tendency to try new things and engage in imaginative and intellectual activities, were found to be associated with a lower risk of developing AD. More recently, these data were substantiated both regarding the risk of dementia (neuroticism a risk factor, conscientiousness protective), cognitive impairment in non-demented individuals; and conversion from such cognitive impairment to dementia (additional risk in individuals with low conscientiousness) (Terracciano et al., 2017).

Personality factors were in addition associated with cognitive impairment in non-demented samples. In particular, neuroticism was associated with a greater risk of cognitive impairment, as well as an accelerated decline in memory and executive functions (Crowe et al., 2006; Caselli et al., 2016). The risk may be heightened in individuals who are simultaneously high in extraversion (a tendency to social interaction) (Crowe et al., 2006). However, a recent study that looked at the longitudinal relationship between personality and cognitive performance across a 20-year follow-up showed that lower neuroticism was associated with better performance in crystallized and fluid abilities at baseline, but not with longitudinal cognitive decline. In this study, higher extraversion was associated with less decline in processing speed longitudinally (Wettstein et al., 2019). Similarly, conscientiousness and openness have been associated with reduced cognitive decline, even in individuals carrying the *APOE*-ε4 allele, the major genetic risk factor for AD (Caselli et al., 2016).

Furthermore, associations between personality traits and brain structure have been shown during aging. For example, Zufferey et al. (2017) showed smaller medial temporal lobe volumes, a region implicated in both AD and age-related cognitive decline (Jack et al., 1998; Krasuski et al., 1998) in individuals with higher neuroticism, regardless of whether they had mild cognitive impairment or not (Zufferey et al., 2017). Interestingly, conscientiousness was negatively associated with total cortical volume in both cross-sectional and longitudinal estimates, as well as with accelerated decline in right medial prefrontal volume, whereas medial temporal regions were better preserved in individuals with AD with high openness (Giannakopoulos et al., 2020b, 2022). Similarly, inferior parietal and right dorsolateral regions were better preserved in healthy individuals with high openness (Taki et al., 2013).

Personality factors such as neuroticism and conscientiousness are tightly linked with neuropsychiatric disorders, depression, anxiety disorders and other psychopathological conditions (Kotov et al., 2010; Andersen and Bienvenu, 2011; Jeronimus et al., 2016). The nature of this relationship has been highly debated. More specifically, personality traits can be viewed as vulnerability factors for mental disorders, be manifestations of the same underlying process, or share the same causes (Jeronimus et al., 2016). Moreover, negative personality traits such as neuroticism have been found to have a substantial genetic overlap with psychiatric disorders such as depression (The Brainstorm Consortium et al., 2018). In common with personality factors, neuropsychiatric symptoms (NPS) such anxiety, depression, or apathy, which are common in AD (Zhao et al., 2016), have also been shown to be risk or predictive factors for future development of AD in non-demented individuals with and without cognitive impairment, as well as in individuals with clinically diagnosed mild cognitive impairment (Peters et al., 2013; Forrester et al., 2016; Acosta et al., 2018; Liew, 2019). For example, in individuals with mild cognitive impairment, the presence of episodes of elevated depressive symptoms increases the risk of dementia (all causes). Also, and their repetition further increases this risk (Dotson et al., 2010). Comparable results were shown in cognitively normal individuals, where the presence of depression significantly increased the risk of future dementia (Saczynski et al., 2010). Similarly, depression and anxiety have been identified as risk factors for the development of mild cognitive impairment in cognitively healthy individuals after a 6-year follow-up period (Burhanullah et al., 2020). Conversely, key neuropathological hallmarks of AD at baseline predicted aggravated apathy and anxiety in cognitively unimpaired individuals over 8 years, further underscoring the link between NPS and neurodegenerative disease (Johansson et al., 2021). However, it can be challenging to determine whether NPSs or personality changes precede neurodegenerative processes or are early symptoms of such processes. In contrast, studies that have measured personality in early life, such as midlife (Johansson et al., 2014) or even adolescence (Chapman et al., 2020), have still found significant associations with dementia 38–54 years later, albeit psychological distress, a NPS, mediated the association in the midlife sample. It is therefore possible that personality traits established early in life predispose individuals to poorer mental health, which in turn increases the risk of neurocognitive declines during aging.

Previous studies on personality and neurocognitive aging, especially longitudinal ones, were mainly based on personality assessments from Costa and McCrae’s 5-factor model: the BIG-FIVE (Costa Jr and McCrae, 2008). By contrast, few studies were based on the Temperament and Character Inventory (TCI) from Cloninger’s psychobiosocial theory of personality (Cloninger et al., 1993). This inventory distinguishes 4 temperament factors: Novelty Seeking, Harm Avoidance, Reward Dependence, Persistence, and three-character factors: Self-Directedness, Cooperativeness, Self-Transcendence. Some of these factors have been associated with a greater risk of mental disorders and psychiatric symptoms in students, adults, and older adults (Margetić et al., 2011; Izci et al., 2014; Norberg et al., 2015; Komasi et al., 2022). Harm avoidance has also been associated with an increased incidence of AD and faster cognitive decline (Wilson et al., 2011). Several of Cloninger’s TCI factors correlate significantly with 5-factor traits. For instance, neuroticism is positively correlated with harm-avoidance (*r* = 0.55) and negatively correlated with self-directedness (*r* = −0.48), while extraversion is negatively correlated with harm-avoidance (*r* = −0.6) (De Fruyt et al., 2000; Capanna et al., 2012). More recently, a study focused on identifying TCI items that were specifically associated with increased risk for Axis I psychiatric disorders (Dell’Orco et al., 2018). As part of this work, the authors identified and validated four new factors: *Optimism* (OTT: optimism about the future, high confidence in one’s own resources and determination), *Closeness to Experience* (CE: avoidance of new stimuli, high anxiety, pessimistic anticipation, low reward seeking), *Tendence to Liabilities* (TL: feeling of inability to change their own reality, low autonomy, low sense of the value of their existence), and *Fantasy of Superiority* (FS: narcissistic fantasy of being more intelligent, attractive and stronger than others, fear of the passage of time and the weakening of the body). Analyses of these new subscales demonstrated good internal consistencies and predictive validity with regard to psychiatric disorders in an external verification sample: OTT being a protective factor, while the other three factors were factors for higher risk of psychiatric disorders (Dell’Orco et al., 2018). Importantly, the revised TCI factors explained more variance in psychiatric symptoms than the original TCI personality factors.

Given the established links between personality traits and NPS, and their relationships to age-related cognitive decline, the objectives of this study were to examine the revised TCI factors from Dell’Orco et al. (2018), considered as pre-existing characteristics and potential risk factors of: (1) NPS in aging; (2) aging-related cognitive change across 25 years, as well as (3) brain gray and white matter structure change across 8 years in a population-based sample. Analyses were stratified on developing Alzheimer’s-like dementia or not, in order to elucidate the effect of neurodegenerative disease on observed associations. A secondary objective was to estimate the risk of AD dementia underpinned by the revised personality factors of the TCI.

It was expected that participants with higher CE, TL, and FS factors would have higher NPS with increasing severity during the follow-up, whereas participants with higher OTT would be more preserved against NPS. With regard to cognition, CE, TL and FS factors were expected to be associated with poorer and more rapidly declining fluid cognition, particularly for age-sensitive functions such as episodic memory and executive function (verbal fluency tests) whereas weaker or no associations were expected for crystallized cognition (vocabulary). On the contrary, OTT would be associated with lower NPS and better cognitive preservation. The neuroimaging analyses focused on regions known to be associated with personality, including frontal, temporal and cingulate structures. It was expected that higher CE, TL and FS factors would be associated with greater volume loss and white matter integrity reductions over time, while the OTT factor would be associated with better structural integrity.

## 2 Methods

### 2.1 Study sample

The participants were part of the Betula study, recruited from the municipality of Umeå on the Northeast coast of Sweden, and randomly sampled from the population register, stratified by age and sex (for a detailed description of the study, see Nilsson et al., 1997, 2004; Nyberg et al., 2020). The age cohorts were divided into 5-year intervals (25, 30, 35, … 80, 85, 95 years) and the number of men and women selected for inclusion was proportional to the male-to-female ratio in each age cohort in the general population. Participants with dementia, non-native Swedish speakers, people with severe hearing or visual impairment, or congenital or acquired intellectual disability were excluded and replaced by another individual from the population registry of the same age and sex. There were no exclusion criteria pertaining to psychiatric disorders, but it is possible that individuals with severe mental illness opted not to participate (see Nilsson et al., 1997). The first wave of data collection started in 1988 and in total six main waves (W1–W6) have been conducted, with the sixth wave completed in 2014. In addition, a seventh wave of testing (W7) was conducted in 2017 for participants returning for a third follow-up neuroimaging (MRI/fMRI) and a limited set of health and cognitive assessments.

In the Betula study, the same diagnostic criteria for dementia were applied throughout the study period (1988–2017). Sufficient information was obtained to apply systems based on clinical criteria, namely the core criteria of the DSM-IV classification for dementia (American Psychiatric Association, 1998) (for detailed descriptions of these procedures, see Nyberg et al., 2020). Participants diagnosed with AD had an insidious onset and progressive cognitive decline and other symptoms typically attributable to clinical AD. Disease onset was defined as the year when clinical symptoms became severe enough to interfere with social functioning and instrumental activities of daily living, i.e., when the basic criteria for dementia were met (McKhann et al., 2011).

### 2.2 Inclusion and exclusion criteria

Only participants who were cognitively healthy or developing Alzheimer dementia were included were included in the analyses. Participants with a neurological or cognitive impairment history, or developing another type of dementia condition (vascular, Lewy body, Parkinson, mixed, other) were excluded. Similarly, participants with missing genetic (*APOE* status: carriers or noncarriers of at least one ε4 allele), personality (TCI), cognition, NPS, or imaging data were excluded from each analysis independently (i.e., for cognition analyses, only participants with missing cognitive data were excluded). Furthermore, outliers beyond ±3 SD from the mean in any neuropsychiatric, cognitive, or neuroimaging variable were excluded. No exclusions were made for mental disorders. The information for each subsample is summarized in Table 1.

**Table 1:** Samples characteristics for every analysis group.

Analysis	Samples	*N* at baseline	Observations	Study waves	Mean follow-up (Min-Max)
Neuropsychiatric	Full sample (CES-D)	1,166	3,174	3–6	8.5 years (0–15)
	Full sample (PHQ-9)	657	1,071	5–6	3 years (0–5)
	Full sample (PSQ)	948	2058	4–6	6 years (0–10)
Sleep (KSQ)	Full sample	445	625	5–6	2 years (0–5)
Cognitive	Full sample	1,286	4,848	1–6	14 years (0–25)
	HC Only	1,145	4,413	1–6	14.5 years (0–25)
Structural MRI	Full sample	235	414	5–7	4 years (0–10)
	HC Only	216	411	5–7	4.5 years (0–10)
DTI	Full sample	228	436	5–7	4.5 years (0–10)
	HC Only	211	410	5–7	4.5 years (0–10)

Full sample, participants with or without conversion to dementia at follow-up; HC, Healthy Controls without conversion to dementia at follow-up; CES-D, Center for Epidemiological Study – Depression Scale; PHQ-9, Patient Health Questionnaire 9 items; PSQ, Perceived Stress Questionnaire; KSQ, Karolinska Sleep Questionnaire; DTI, Diffusion tensor imaging; baseline, refers to the wave where the first evaluations of the model were performed (5th for retained neuropsychiatric data and imagery, 1st for cognitive data).

### 2.3 Personality data

Personality factors were assessed with the Swedish version of TCI. This version comprises 238 items, each consisting of a statement to which the participant must answer “true” or “false.” For each participant, the first personality measure was retained. The TCI was introduced at Wave 2, and if data from this wave was missing, data from the third Wave was instead retained (if available), given that personality is considered to be relatively stable across development and aging (Brändström et al., 2008). Data were available for 1,433 participants assessed at Wave 2, and 195 assessed at Wave 3.

The personality factors used were derived from the new factors determined by the analyses of Dell’Orco et al. (2018). Cloninger’s original factors were not analyzed, instead, the new factors are based on a recombination of the items that underlie Cloninger’s factors. The dimension “Optimism” (OTT) describes subjects who are clearly optimistic about the future, have high confidence in their own resources, and are determined to act. The “Closeness to Experience” (CE) dimension describes subjects who avoid novel stimuli, have marked anxiety, pessimistic anticipatory tendency, and habitual behaviors and reflexivity, low reward seeking, and refusal of unusual activities. The dimension “Tendence to Liabilities” (TL) describes subjects who see themselves as unable to change their own reality, with low autonomy and with a low sense of the value of their existence. The dimension “Fantasy of Superiority” (FS) describes subjects who narcissistically fantasize about being smarter, more attractive and stronger than anyone else and who fear the passage of time and the weakening of the body. Before analyses, the variables for each factor were z-transformed.

### 2.4 Neuropsychiatric data

Neuropsychiatric data were extracted from waves 3 to 6 depending on the questionnaire used and consisted of assessments of depression, stress, and sleep. For a description of availability of data from different study waves, see Table 1.

Depression was assessed by the Patient Health Questionnaire-9 (PHQ-9) (Kroenke et al., 2001). This is a self-report consisting of 9 questions designed to screen for the presence and severity of depression in adult patients. Each question assesses the frequency of problems experienced by the individuals in the past 2 weeks. This frequency is rated from 0 to 3 (0: not at all, 1: a few days, 2: at least half the days, 3: almost every day). The questionnaire shows good internal consistency across different validation studies (Cronbach’s α ~ 0.9) (Hansson et al., 2009; Beard et al., 2016).

Depression was also assessed by the Center for Epidemiologic Studies-Depression scale (CES-D) (Radloff, 1977). This is a self-report questionnaire of 20 items designed to screen for the presence and severity of depression in adults. Each question assesses the frequency of affect experienced by individuals during the past week. This frequency is rated from 0 to 3 (0: never/very rarely, 1: occasionally, 2: often, 3: frequently/permanently). Four of the 20 items are reversed. The scale has a good internal consistency in the general and the patient populations (Cronbach’s α = 0.85–0.9) (Radloff, 1977).

Stress was assessed using the Perceived Stress Questionnaire (Levenstein et al., 1993; Rönnlund et al., 2015). This 30-item questionnaire is a self-report of the frequency of stressful feelings and experiences rated from never/very rarely (coded as 1), occasionally (2), often (3), frequently/always (coded as 4). Eight of the 30 items are reversed. A Cronbach’s alpha value of 0.90 has been reported in the Swedish population (Bergdahl and Bergdahl, 2002).

Sleep was assessed using the Karolinska Sleep Questionnaire (Kecklund and Åkerstedt, 1992). In addition to assessing the sleep characteristics of individuals (need, sufficiency, quality, schedule…), the questionnaire has 4 subscales: sleep apnea consisting of 3 items, sleepiness consisting of 5 items, sleep quality consisting of 4 items, and non-restorative sleep consisting of 3 items. Each item focuses on the frequency of experiences based on the last 3 months and is scored from 0 to 5 (0: never, 1: rarely, 2: sometimes, 3: often, 4: most of the time, 5: always). The Cronbach’s alpha range from 0.73 to 0.84 for sleep quality, from 0.71 to 0.82 for non-restorative sleep, from 0.75 to 0.83 for sleep apnea, and from 0.84 to 0.87 for sleepiness (Nordin et al., 2013). The sum of the scores was used as the total score of the questionnaire.

### 2.5 Neuropsychological data

The cognitive battery used in this study includes a composite of five episodic memory scores (including free recall, cueing and recognition tasks for different types of material: words, sentences, actions), vocabulary from the SRB (Dureman, 1960), the WAIS-R Block Design test, and a composite verbal fluency score calculated on the basis of the average of the z-scores of different fluency tests (words starting with letter A, 5-letter and starting with letter M, occupations starting with letter B). For full description of the cognitive tests and testing procedures, see Nyberg et al. (2020).

### 2.6 Health data

At the first visit, participants were asked about their past and present illnesses/conditions (e.g., cardiovascular disease, high blood pressure [HBP], diabetes). At the follow-up visits, they were asked about these conditions over the past 5 years (since the year of the previous visit). For each condition of interest (cardiovascular disease, HBP, diabetes), it was recorded whether the participants had encountered these conditions (1 = yes, 0 = no) at any point before or during the study period. These data were used as additional controls in statistical models considering them as risk factors for Alzheimer’s disease and dementia (Livingston et al., 2017).

### 2.7 MRI and DTI data

MRI acquisition was performed on the same General Electric 3 T scanner equipped with a 32-channel head coil at all three time-points (W5-W7). Head movement was minimized using cushions inside the head coil for all imaging sequences. Invitations to participate in the imaging substudy were offered to participants in cohorts 1, 3, and 6 with complete health and cognitive data at visit 5, without contraindications to MRI, severe neurological disorders, or motor/visuospatial deficits. Participants were selected via stratification by age and sex.

The T1-weighted images were acquired with a 3D fast spoiled gradient echo sequence (180 slices with a 1 mm thickness; TR: 8.2 ms, TE: 3.2 ms, flip angle: 12°, field of view: 250 × 250 mm). For the extraction of gray and white matter volumes, we used the longitudinal pipeline of Freesurfer v. 6.0 (Martinos Center for Biomedical Imaging, Charlestown, MA, United States) where regional cortical thicknesses and volumetric measures were estimated (Fischl et al., 2002; Reuter et al., 2012; Iglesias et al., 2016). The software is well documented and available for download online.

Diffusion-weighted images were acquired with a spin-echo-planar T2-weighted sequence as follows: 64 slices, TR = 8,000 ms, TE = 84.4 ms, flip angle = 90°, FOV = 250 × 250 mm, b = 1,000 s/mm^2^, 32 independent directions, and six b = 0 images. Three sequence repetitions were acquired at W5 and W6, whereas W7 only included one repetition. To allow for consistency between waves, only the first repetition at each wave was used. Diffusion data were preprocessed using the University of Oxford’s Center for Functional Magnetic Resonance Imaging of the Brain (FMRIB) Software Library (FSL) package^1^ and the fractional anisotropy (FA) was extracted. More details are available in previous studies using Betula imaging data (Avelar-Pereira et al., 2020; Pedersen et al., 2021).

As structural variables of interest, the following gray and white matter volumes were extracted: frontal, temporal, and cingulate gray and white matter, total cortical gray matter, as well as bilateral hippocampal volumes. The Table 2 describes the structures used in the calculation of total lobar volumes.

**Table 2:** Details of the structures included in the calculation of lobar volumes.

Lobes	Structures
Frontal	Frontal pole, paracentral gyrus, pars opercularis/triangularis/orbitalis gyri, caudal/rostral middle gyri, superior gyrus, lateral/medial orbitofrontal gyri, precentral gyrus
Temporal	Superior temporal sulcus, entorhinal gyrus, fusiform gyrus, inferior/middle/superior temporal gyri, parahippocampal gyrus, temporal pole, temporal transverse
Cingulate	Caudal/rostral anterior, posterior, isthmus

Similarly, diffusion variables of interest, based on the FA of white matter networks, involved the fornix, cingulate cingulum, cingulum-hippocampal networks, and the uncinate tracts. These structures were selected because of their relationship with personality factors from both structural MRI and DTI studies (Kaasinen et al., 2005; Gardini et al., 2009; DeYoung et al., 2010; Jackson et al., 2011; Xu and Potenza, 2012; Coutinho et al., 2013; Liu et al., 2013; Taki et al., 2013; Lu et al., 2014; Bauer et al., 2016; Li et al., 2017; Zufferey et al., 2017; Prillwitz et al., 2018; Sanjari Moghaddam et al., 2020; Giannakopoulos et al., 2020a,b, 2022).

The exclusion of outliers from the MRI/DTI data was based on the following procedure. Each structure’s volume or FA was standardized to z-scores according to the formula: (subject volume or FA - baseline group mean volume or FA)/baseline group SD volume or FA. Z-scores higher than |3| were excluded. All subsequent analyses were performed using the z-score variables.

### 2.8 Genotyping

Genotyping for the Apolipoprotein E (*APOE*) gene (ε2, ε3, ε4 alleles) was performed by polymerase chain reaction, and was available for the subsamples described in Table 1 (*N*_max_ = 1,286) (see Sundström et al., 2004).

### 2.9 Statistical analysis

Statistical analyses were performed on the open-source web application Jupyter Notebook (version 6.3.0) using scripts written in Python language (version 3.9.5). For the neuropsychiatric and cognitive models, the continuous data were standardized into z-scores according to the formula: (subject scores - baseline group mean)/baseline group SD.

First, correlations were performed using Pearson’s tests to describe associations between the personality factors. In case of significant correlations, the subsequent linear mixed-effects regression models considered each personality factor independently in order to avoid multicollinearity. These correlations were performed with the scipy.stats.pearsonr function.

In a second step, we used linear mixed-effects regression models to test the main and time-interaction effects of personality factors with acceptable internal consistency on NPS. These models were used as a step towards validating personality traits. For each model, age at baseline, quadratic age at baseline, time (years from the baseline), sex, *APOE* status, interaction between time and *APOE* status, personality factors, and interaction between time and personality factors were considered fixed factors (time, personality factors, and their interaction terms as factors of interest, the others as covariates).

In a last step, linear mixed-effects regression models were used to test the main and time-interaction effects of validated personality factors on cognitive performance, and brain structures/diffusivity. For each model, age at baseline, quadratic age at baseline, time (years from the baseline), sex, years of education, *APOE* status, interaction between time and *APOE* status, personality factors, and interaction between time and personality factors were considered fixed factors (time, personality factors, and interaction terms as factors of interest, the others as covariates). Additionally, the estimated total intracranial volume (eTIV) was used as a covariate in hippocampal volume regression models. Individual participants were considered as random factors. All models included random effects for intercepts and slopes. These models were generated with the statsmodel package and the functions statsmodel.api and statsmodel.formula.api.mixedlm. Despite data preparation steps to normalize the distributions, they did not meet the residuals normality criterion for linear mixed models. However, recent work has demonstrated the robustness of these models even when this application condition was violated (Schielzeth et al., 2020).

Each regression model was estimated according to the form:

Dependent variables ~ age + age^2^ + sex + APOE + education + (eTIV) + time + APOE*Time + Personality + Personality*time + random intercept + random slope.

In a supplementary control analysis, health conditions (cardiovascular disease, HBP, diabetes) and their interactions with time were added as covariates in cognitive and MRI models.

NPS, cognitive performance, and brain structures were used as dependent variables in separate models. In order to enhance convergence of the model matrices, Powell’s method for fitting data was applied.

A Cox proportional hazards regression was used to predict the risk of Alzheimer’s type dementia based on demographics, *APOE* status, and revised personality factors. Dementia diagnosis was treated as the failure event. Years from the baseline (first measure wave) was used as the time scale. The last available visit was used for censoring participants not developing dementia, whereas the conversion visit was used for participants developing dementia. Finally, age, sex, years of education, *APOE* status (*APOE*-ε4 carriers or not), and scores on the two validated revised TCI personality factors (CE, TL) were used as covariates. This analysis was performed using the lifelines.CoxPHFitter package.

For the cognitive and cerebral models, a False Discovery Rate correction was applied by considering the number of dependent variables (4 for cognitive, 15 for brain volumes, 7 for FA), the number of fixed interest factors (3 per model: main effect of time, main effect of personality factor and its interaction with time), and the number of validated personality factors (2) as repetition factors. The corrections were applied using the package statsmodel, and the function stats.multitest.fdrcorrection with a Benjamini/Hochberg method and a Family-wise error rate of 0.05. The results tables display the adjusted *p*-values (*p*-adj), which are considered significant if they are <0.05.

## 3 Results

### 3.1 Demographic characteristics

The main sample selected comprised 1,149 healthy participants at baseline not converting to AD dementia during follow-up and 137 participants converting during follow-up. These two subgroups differed in age, years of education, Mini Mental State Evaulation (MMSE) score, sex and *APOE* status distribution. These descriptive data are summarized in the Table 3.

**Table 3:** Demographic characteristics of the total sample retained at baseline.

	HC	AD	Statistic *T*/*χ*^2^	*p*-value
N	1,149	137	–	–
Age M (SD)	48.13 (8.30)	56.84 (5.33)	−4.49	<0.001
Education M(SD)	12.23 (3.85)	8.89 (3.46)	3.65	<0.001
MMSE M (SD)	27.98 (1.65)	27.42 (1.87)	1.39	0.166
Women %	50.46	84.21	6.69	0.010
*APOE*-ε4%	25	52.63	5.41	0.02

HC, participant who not converted in any dementia type; AD, participants who converted to AD dementia type during the follow-up. M, mean; SD, standard deviation; MMSE, Mini Mental State Evaluation.

The subsample for MRI analysis included 216 healthy participants at baseline who did not convert to AD dementia during follow-up, and 19 participants who converted during follow-up. The descriptive data are summarized in the Table 4.

**Table 4:** Demographic characteristics of the MRI subsample retained at baseline.

	HC	AD	Statistic T/Chi^2^	*p*-value
N	216	19	–	–
Age M (SD)	48.13 (13.55)	66.93 (9.16)	−9.33	<0.001
Education M (SD)	10.47 (3.92)	8.00 (2.81)	7.17	<0.001
MMSE M (SD)	27.97 (1.66)	27.40 (2.00)	3.67	<0.001
Women %	51.52	75.91	28.35	<0.001
*APOE*-ε4%	27.50	54.74	41.65	<0.001

HC, participant who not converted in any dementia type; AD, participants who converted in AD dementia type during the follow-up. M, mean; SD, standard deviation; MMSE, Mini Mental State Evaluation.

### 3.2 Internal consistency and intercorrelations of revised TCI factors

For verification purposes, the internal consistency of the factors in our sample as well as the correlation between measurement times 2 and 3 (W2–W3) were tested. The data indicate that two of the factors have acceptable internal consistency (CE and TL) in our sample, and two have questionable internal consistency (OTT and FS). Only CE and TL were retained for further analyses. The data are summarized in Table 5, which also shows the relative stability of the constructs across 5 years (*r* = 0.67, W2–W3).

**Table 5:** Internal consistency and correlation between revised TCI factors in whole sample.

TCI factor	Cronbach’s α	OTT	CE	TL	FS	Mean ± SD
OTT	0.61	*r*_w2–w3_ = 0.62				3.27 ± 1.62
CE	0.78	*r*_w2–w2_ = −0.31	*r*_w2–w3_ = 0.67			2.99 ± 2.75
TL	0.76	*r*_w2–w2_ = −0.20	*r*_w2–w2_ = 0.62	*r*_w2–w3_ = 0.67		2.60 ± 2.62
FS	0.67	*r*_w2–w2_ = 0.02	*r*_w2–w2_ = 0.40	*r*_w2–w2_ = 0.44	*r*_w2–w3_ = 0.69	1.96 ± 1.91

OTT, optimism; CE, Closeness to Experience; TL, Tendence to Liabilities; FS, Fantasy of Superiority. Cronbach’s Interpretation: >0.70 is acceptable, >0.60 is questionable (Gliem and Gliem, 2003). *r*, Pearson’s correlation coefficient; *r*_w2–w3_, correlation between assessments from waves 2 and 3; *r*_w2–w2_, correlation between assessments from wave 2. SD, standard deviation. Every coefficient is significant at *p* < 0.001 except for OTT*FS correlation (*p* = 0.591).

### 3.3 Neuropsychiatric trajectories related to revised TCI factors

Controlling for age, sex, and *APOE* status, CE showed a positive cross-sectional association with CES-D (depression), and a positive longitudinal association with PSQ (stress). TL also showed positive cross-sectional associations with CES-D, PSQ, and KSQ (sleep), as well as a positive longitudinal association with PHQ-9 (depression). Thus, the higher the CE and TL were, the higher the depression, stress and sleep disturbances were, and the faster the longitudinal progression of depression and stress symptoms. Assessments with CES-D, PHQ, PSQ and KSQ were made, respectively, 10.01 ± 5.60; 16.43 ± 2.98; 13.27 ± 4.29; and 16.53 ± 2.97 years after the personality assessment. The neuropsychiatric and sleep results are presented in Table 6.

**Table 6:** Effects of time, personality factors and their interactions on neuropsychiatric symptoms.

Dependent	Fixed factors	Coef.	Std. Err.	*p*-value	[0.025	0.975]
CES-D	Time	0.001	0.003	0.835	−0.005	0.006
	CE	0.249	0.040	**<0.001** ^***^	0.171	0.328
	Time*CE	0.003	0.003	0.298	−0.003	0.008
	Time	0.001	0.003	0.859	−0.005	0.006
	TL	0.237	0.042	**<0.001** ^***^	0.155	0.320
	Time*TL	0.003	0.003	0.235	−0.002	0.009
PHQ-9	Time	0.010	0.008	0.187	−0.005	0.026
	CE	−0.038	0.140	0.787	−0.312	0.236
	Time*CE	0.014	0.007	*0.055* ^†^	−0.000	0.028
	Time	0.010	0.008	0.221	−0.006	0.025
	TL	−0.062	0.149	0.678	−0.354	0.230
	Time*TL	0.016	0.008	**0.042** ^*^	0.001	0.031
PSQ	Time	−0.028	0.004	**<0.001** ^***^	−0.037	−0.020
	CE	0.073	0.068	0.281	−0.060	0.207
	Time*CE	0.012	0.004	**0.002** ^**^	0.004	0.020
	Time	−0.029	0.004	**<0.001** ^***^	−0.037	−0.021
	TL	0.145	0.070	**0.038** ^*^	0.008	0.283
	Time*TL	0.006	0.004	0.141	−0.002	0.014
KSQ	Time	−0.006	0.011	0.552	−0.028	0.015
	CE	0.159	0.210	0.448	−0.252	0.570
	Time*CE	0.002	0.011	0.868	−0.019	0.023
	Time	−0.010	0.011	0.378	−0.031	0.012
	TL	0.497	0.220	**0.024** ^*^	0.066	0.928
	Time*TL	−0.018	0.011	0.102	−0.040	0.004

CES-D, Center for Epidemiologic Studies-Depression Scale; PSQ, Perceived Stress Questionnaire; PHQ-9, Patient Health Questionnaire-9 items; KSQ, Karolinska Sleep Questionnaire; CE, Closeness to Experience; TL, Tendence to Liabilities. Models are adjusted for age, sex, and *APOE* status of participants. Coefficients are expressed in standard deviation units (z-scores). Bold font indicates *p*-values <0.05. Italicized font indicates *p*-values <0.1 but larger than 0.05. ^†^*p* < 0.10; ^*^p < 0.05; ^**^p < 0.01; ^***^p < 0.001.

### 3.4 Cognitive trajectories related to revised TCI factors

Controlling for age, sex, education, *APOE* status, and adjusting the models with an FDR correction, both CE and TL personality factors showed significant effects on cross-sectional and longitudinal cognitive outcomes.

The unadjusted and adjusted cognitive outcomes are summarized in Table 7. We report results for the full samples, as well as a healthy subset of non-AD converting participants, in order to determine whether potential personality-related effects on cognitive performance are driven by pre-clinical AD-related declines.

**Table 7:** Effects of time, personality factors and their interactions on cognitive performance.

Dep.	Fixed factors	Full sample						HC Sample					
		Coef.	Std. Err.	*p*-value	[0.025	0.975]	*p*_fdr	Coef.	Std. Err.	*p*-value	[0.025	0.975]	*p*_fdr
Mem	Time	−0.017	0.002	**<0.001** ^***^	−0.020	−0.014	**<0.001** ^***^	−0.013	0.001	**<0.001** ^***^	−0.016	−0.010	**<0.001** ^***^
	CE	−0.044	0.024	*0.059* ^†^	−0.091	0.002	*0.070* ^†^	−0.048	0.025	*0.054* ^†^	−0.096	0.001	*0.093* ^†^
	Time*CE	−0.006	0.002	**<0.001** ^***^	−0.009	−0.003	**0.002** ^**^	−0.004	0.002	**0.006** ^**^	−0.007	−0.001	**0.022** ^*^
	Time	−0.017	0.002	**<0.001** ^***^	−0.020	−0.013	**<0.001** ^***^	−0.013	0.001	**<0.001** ^***^	−0.016	−0.010	**<0.001** ^***^
	TL	−0.013	0.023	0.573	−0.059	0.033	0.573	−0.036	0.025	0.148	−0.084	0.013	0.170
	Time*TL	−0.005	0.002	**0.001** ^**^	−0.008	−0.002	**0.004** ^**^	−0.003	0.002	*0.071* ^†^	−0.006	0.000	0.107
Voc	Time	−0.004	0.001	**0.003** ^**^	−0.006	−0.001	**0.007** ^**^	−0.003	0.001	**0.013** ^*^	−0.005	−0.001	**0.034** ^*^
	CE	−0.063	0.023	**0.007** ^**^	−0.109	−0.017	**0.013** ^*^	−0.058	0.024	**0.018** ^*^	−0.105	−0.010	**0.036** ^*^
	Time*CE	−0.003	0.001	**0.012** ^*^	−0.005	−0.001	**0.018** ^*^	−0.002	0.001	*0.062* ^†^	−0.005	0.000	*0.099* ^†^
	Time	−0.003	0.001	**0.003** ^**^	−0.006	−0.001	**0.008** ^**^	−0.003	0.001	**0.017** ^*^	−0.005	−0.000	**0.036** ^*^
	TL	−0.063	0.023	**0.006** ^**^	−0.108	−0.018	**0.013** ^*^	−0.075	0.024	**0.002** ^**^	−0.123	−0.027	**0.010** ^*^
	Time*TL	−0.003	0.001	**0.012** ^*^	−0.005	−0.001	**0.018** ^*^	−0.002	0.001	*0.089* ^†^	−0.004	0.000	0.118
Block	Time	−0.028	0.001	**<0.001** ^***^	−0.030	−0.025	**<0.001** ^***^	−0.027	0.001	**<0.001** ^***^	−0.029	−0.024	**<0.001** ^***^
	CE	−0.066	0.025	**0.008** ^**^	−0.114	−0.018	**0.013** ^*^	−0.055	0.026	**0.038** ^*^	−0.107	−0.003	*0.071* ^†^
	Time*CE	−0.001	0.001	0.307	−0.004	0.001	0.334	−0.001	0.001	0.307	−0.004	0.001	0.320
	Time	−0.028	0.001	**<0.001** ^***^	−0.030	−0.025	**<0.001** ^***^	−0.027	0.001	**<0.001** ^***^	−0.029	−0.024	**<0.001** ^***^
	TL	−0.055	0.025	**0.024** ^*^	−0.104	−0.007	**0.032** ^*^	−0.046	0.026	*0.084* ^†^	−0.098	0.006	0.118
	Time*TL	−0.002	0.001	0.172	−0.004	0.001	0.197	−0.002	0.001	0.227	−0.004	0.001	0.247
Flu	Time	−0.005	0.001	**<0.001** ^***^	−0.007	−0.002	**<0.001** ^***^	−0.003	0.001	**0.009** ^**^	−0.006	−0.001	**0.028** ^*^
	CE	−0.057	0.021	**0.006** ^**^	−0.098	−0.017	**0.013** ^*^	−0.053	0.022	**0.016** ^*^	−0.096	−0.010	**0.036** ^*^
	Time*CE	−0.003	0.001	**0.033** ^*^	−0.005	−0.000	**0.041** ^*^	−0.002	0.001	0.121	−0.005	0.001	0.153
	Time	−0.005	0.001	**<0.001** ^***^	−0.007	−0.002	**<0.001** ^***^	−0.003	0.001	**0.006** ^**^	−0.006	−0.001	**0.022** ^*^
	TL	−0.014	0.021	0.504	−0.054	0.027	0.526	−0.015	0.023	0.515	−0.060	0.030	0.515
	Time*TL	−0.003	0.001	**0.014** ^*^	−0.006	−0.001	**0.019** ^*^	−0.002	0.001	0.139	−0.004	0.001	0.167

Dep., dependent variable; Mem, Episodic Memory; Voc., vocabulary; Bloc, bloc design; Flu, verbal fluency; CE, Closeness to Experience; TL, Tendence to Liabilities. Models are adjusted for age, sex, education, and *APOE* status of participants. Coefficients are expressed in standard deviation units (z-scores). Bold font indicates *p*-values <0.05. Italicized font indicates *p*-values <0.1 but larger than 0.05. ^†^*p* < 0.10; ^*^p < 0.05; ^**^p < 0.01; ^***^p < 0.001.

**Figure 1:**
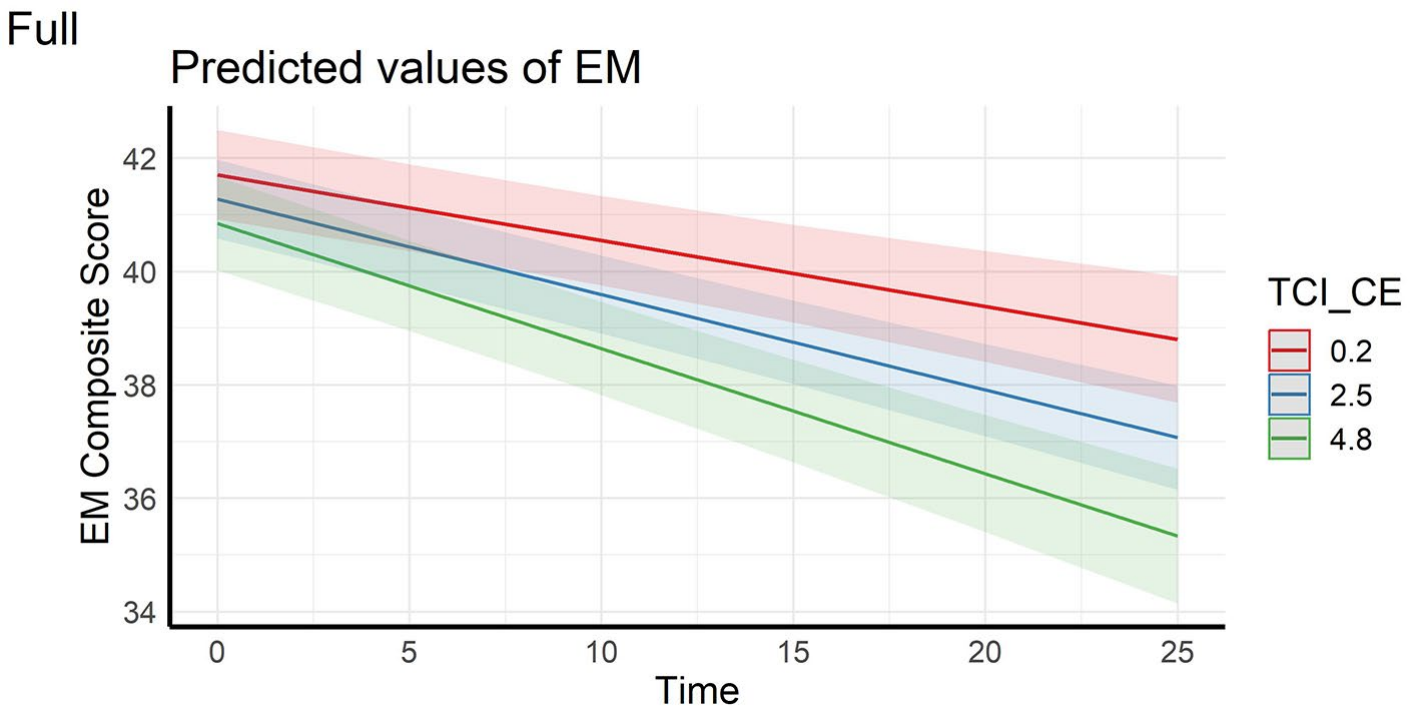
Interaction effect between Closeness to Experience and Time on Episodic memory composite score in the full sample. EM, Episodic Memory; TCI_CE, Closeness to Experience score assessed with Temperament and Character Inventory.

**Figure 2:**
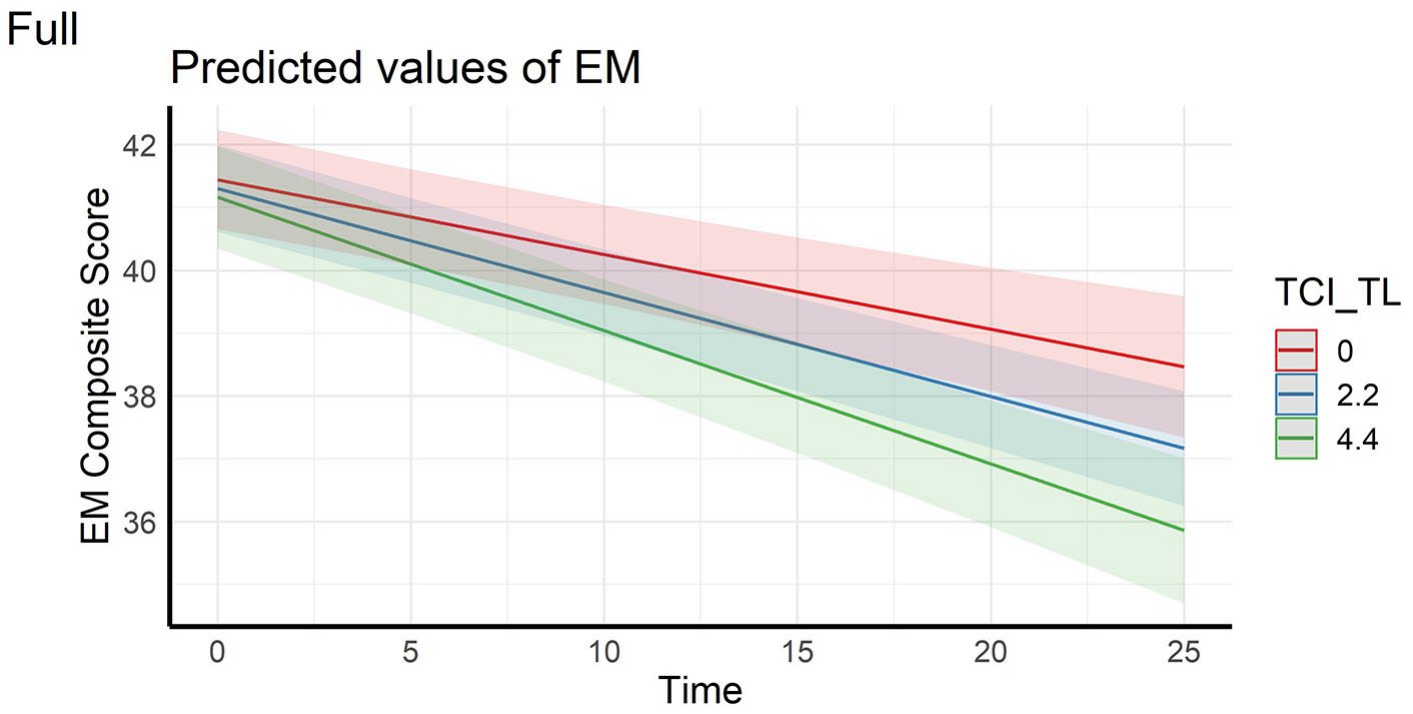
Interaction effect between Tendence to Liabilities and Time on Episodic memory composite score in the full sample. EM, Episodic Memory; TCI_TL, Tendence to Liabilities score assessed with Temperament and Character Inventory.

**Figure 3:**
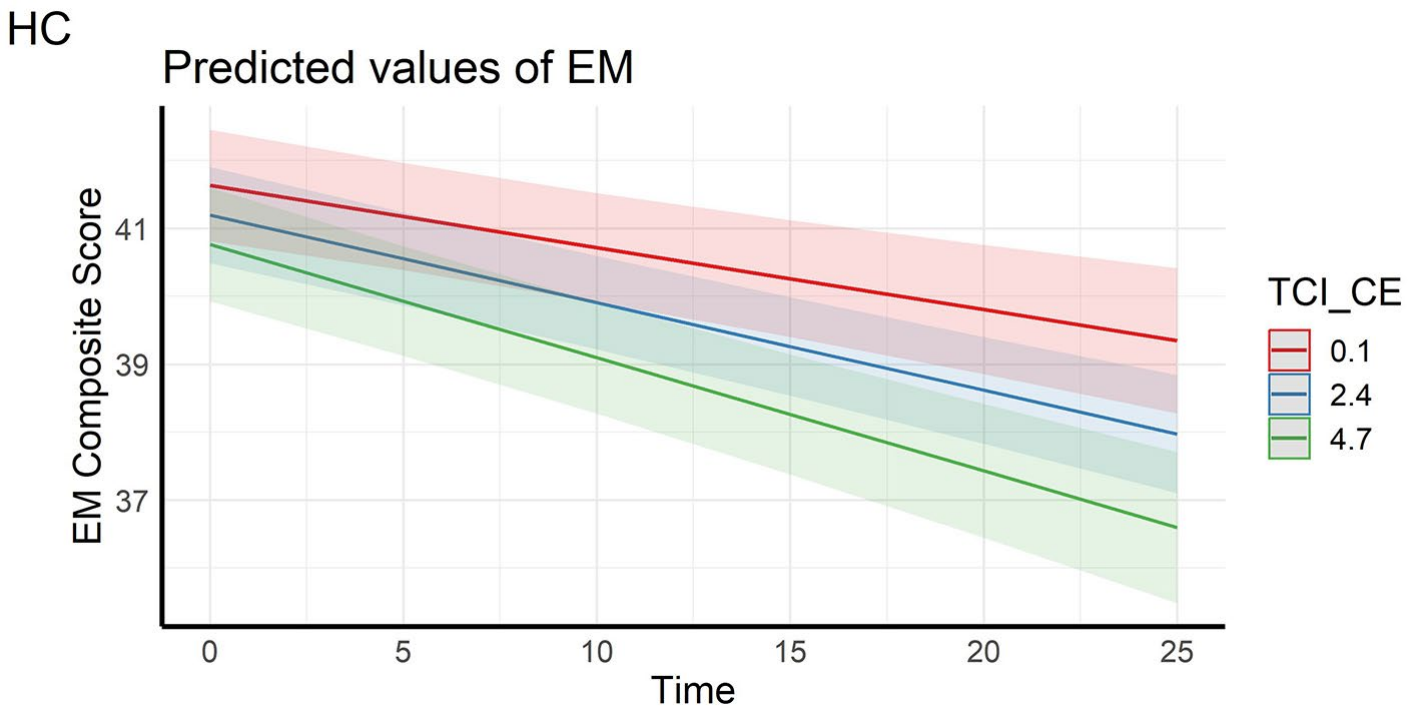
LInteraction effect between Closeness to Experience and Time on Episodic memory composite score in the healthy control subsample. HC, Healthy Control subsample; EM, Episodic Memory; TCI_CE, Closeness to Experience score assessed with Temperament and Character Inventory.

In the full sample, the analyses showed that episodic memory performance decreased over time, and decreased faster with higher scores on personality factors CE and TL (main effect of time and time x personality interaction effects – Figures 1, 2). The same longitudinal effect was observed in HC subsample related to factor CE (Figure 3). These effects were in line with our hypotheses.

Concerning vocabulary performance, higher CE and TL factors were associated with lower performance (cross-sectional main effects) in the full sample, performance decreased over time and significantly more so with higher scores on the CE and TL factors (longitudinal main effects of time and personality x time-interaction effects). In the HC subsample, higher CE and TL factors were only significantly associated with lower baseline performance (cross-sectional main effects). Also, performance decreased over time (main effect of time).

Regarding the block design task, higher CE and TL factors were associated with lower baseline performance (cross-sectional main effects) in the full sample, and performance decreased over time (longitudinal main effect of time). The cross-sectional effect of CE and main effect of time were also observed in HC subsample. No longitudinal interaction effects between time and personality factors on performance were observed.

For verbal fluency performance, higher scores on the CE factor were associated with lower performance (cross-sectional main effect), performance decreased over time and more so for higher the CE and TL scores (longitudinal main effect of time and personality x time interaction effects). This was in line with our hypothesis of higher decline of fluid cognition related to negative personality factors. In the HC subsample, only a cross-sectional effect of CE and a main effect of time were observed.

### 3.5 Brain trajectories related to revised TCI factors

Controlling for age, sex, education, *APOE* status, and adjusting the models with an FDR correction, mainly the CE personality factor showed significant effects on brain characteristics longitudinally.

The unadjusted and adjusted neuroimaging outcomes are summarized in Table 8 (gray matter volumes) and Table 9 (white matter tract’s FA), and Supplementary Table S1 (white matter volumes).

**Table 8:** Effects of time, personality factors and their interactions on gray matter volumes.

Dependent	Fixed factors	Full Sample						HC Sample					
		Coef.	Std. Err.	*p*-value	[0.025	0.975]	*p*_fdr	Coef.	Std. Err.	*p*-value	[0.025	0.975]	*p*_fdr
Hipp L	Time	−0.026	0.003	**<0.001** ^***^	−0.033	−0.019	**<0.001** ^***^	−0.027	0.003	**<0.001** ^***^	−0.033	−0.020	**<0.001** ^***^
	CE	−0.055	0.052	0.293	−0.158	0.047	0.540	−0.056	0.055	0.311	−0.165	0.052	0.596
	Time*CE	−0.008	0.003	**0.004** ^**^	−0.013	−0.002	**0.016** ^*^	−0.006	0.003	**0.023** ^*^	−0.011	−0.001	*0.062* ^†^
	Time	−0.027	0.004	**<0.001** ^***^	−0.034	−0.020	**<0.001** ^***^	−0.027	0.003	**<0.001** ^***^	−0.034	−0.021	**<0.001** ^***^
	TL	−0.030	0.052	0.563	−0.133	0.072	0.814	−0.010	0.058	0.858	−0.124	0.103	0.982
	Time*TL	−0.003	0.003	0.359	−0.008	0.003	0.626	−0.004	0.003	0.170	−0.010	0.002	0.379
Hipp R	Time	−0.029	0.004	**<0.001** ^***^	−0.037	−0.021	**<0.001** ^***^	−0.029	0.004	**<0.001** ^***^	−0.036	−0.022	**<0.001** ^***^
	CE	−0.085	0.053	0.111	−0.189	0.020	0.267	−0.066	0.052	0.205	−0.167	0.036	0.418
	Time*CE	−0.006	0.003	**0.033** ^*^	−0.012	−0.001	*0.092* ^†^	−0.007	0.003	**0.008** ^**^	−0.013	−0.002	**0.022** ^*^
	Time	−0.030	0.004	**<0.001** ^***^	−0.038	−0.022	**<0.001** ^***^	−0.030	0.004	**<0.001** ^***^	−0.037	−0.023	**<0.001** ^***^
	TL	−0.002	0.054	0.975	−0.107	0.103	0.996	0.005	0.054	0.924	−0.101	0.111	0.986
	Time*TL	−0.004	0.003	0.198	−0.011	0.002	0.432	−0.005	0.003	0.114	−0.011	0.001	0.274
Cortex L	Time	−0.043	0.002	**<0.001** ^***^	−0.048	−0.039	**<0.001** ^***^	−0.041	0.002	**<0.001** ^***^	−0.045	−0.037	**<0.001** ^***^
	CE	−0.031	0.049	0.528	−0.127	0.065	0.792	−0.020	0.052	0.698	−0.122	0.082	0.938
	Time*CE	0.000	0.002	0.845	−0.003	0.004	0.978	0.001	0.002	0.674	−0.002	0.004	0.938
	Time	−0.043	0.002	**<0.001** ^***^	−0.048	−0.039	**<0.001** ^***^	−0.041	0.002	**<0.001** ^***^	−0.045	−0.037	**<0.001** ^***^
	TL	−0.011	0.049	0.821	−0.107	0.085	0.973	0.001	0.054	0.983	−0.105	0.108	0.996
	Time*TL	0.002	0.002	0.274	−0.002	0.006	0.526	0.002	0.002	0.179	−0.001	0.006	0.381
Cortex R	Time	−0.043	0.002	**<0.001** ^***^	−0.048	−0.039	**<0.001** ^***^	−0.041	0.002	**<0.001** ^***^	−0.045	−0.037	**<0.001** ^***^
	CE	−0.031	0.049	0.528	−0.127	0.065	0.792	−0.020	0.052	0.698	−0.122	0.082	0.938
	Time*CE	0.000	0.002	0.845	−0.003	0.004	0.978	0.001	0.002	0.674	−0.002	0.004	0.938
	Time	−0.043	0.002	**<0.001** ^***^	−0.048	−0.039	**<0.001** ^***^	−0.041	0.002	**<0.001** ^***^	−0.045	−0.037	**<0.001** ^***^
	TL	−0.011	0.049	0.821	−0.107	0.085	0.973	0.001	0.054	0.983	−0.105	0.108	0.996
	Time*TL	0.002	0.002	0.274	−0.002	0.006	0.526	0.002	0.002	0.179	−0.001	0.006	0.381
Frontal L	Time	−0.038	0.002	**<0.001** ^***^	−0.042	−0.033	**<0.001** ^***^	−0.036	0.002	**<0.001** ^***^	−0.040	−0.032	**<0.001** ^***^
	CE	−0.048	0.053	0.365	−0.152	0.056	0.626	−0.035	0.056	0.531	−0.145	0.075	0.864
	Time*CE	−0.000	0.002	0.984	−0.004	0.004	0.996	0.000	0.002	0.984	−0.003	0.003	0.996
	Time	−0.038	0.002	**<0.001** ^***^	−0.042	−0.033	**<0.001** ^***^	−0.036	0.002	**<0.001** ^***^	−0.040	−0.032	**<0.001** ^***^
	TL	−0.017	0.053	0.746	−0.121	0.087	0.918	0.002	0.059	0.973	−0.113	0.117	0.996
	Time*TL	0.002	0.002	0.306	−0.002	0.006	0.555	0.002	0.002	0.282	−0.002	0.005	0.553
Frontal R	Time	−0.039	0.002	**<0.001** ^***^	−0.044	−0.034	**<0.001** ^***^	−0.037	0.002	**<0.001** ^***^	−0.041	−0.033	**<0.001** ^***^
	CE	−0.017	0.053	0.744	−0.121	0.086	0.918	−0.001	0.055	0.991	−0.109	0.108	0.996
	Time*CE	0.000	0.002	0.902	−0.004	0.004	0.995	0.000	0.002	0.828	−0.003	0.004	0.982
	Time	−0.040	0.002	**<0.001** ^***^	−0.044	−0.035	**<0.001** ^***^	−0.037	0.002	**<0.001** ^***^	−0.042	−0.033	**<0.001** ^***^
	TL	−0.007	0.053	0.896	−0.110	0.096	0.995	0.020	0.058	0.731	−0.093	0.133	0.952
	Time*TL	0.002	0.002	0.236	−0.002	0.006	0.482	0.002	0.002	0.347	−0.002	0.005	0.629
Temporal L	Time	−0.037	0.003	**<0.001** ^***^	−0.043	−0.031	**<0.001** ^***^	−0.038	0.003	**<0.001** ^***^	−0.043	−0.032	**<0.001** ^***^
	CE	−0.020	0.049	0.685	−0.116	0.076	0.877	−0.012	0.052	0.814	−0.115	0.090	0.982
	Time*CE	0.002	0.002	0.350	−0.002	0.006	0.623	0.003	0.002	0.131	−0.001	0.007	0.306
	Time	−0.037	0.003	**<0.001** ^***^	−0.043	−0.031	**<0.001** ^***^	−0.037	0.003	**<0.001** ^***^	−0.043	−0.032	**<0.001** ^***^
	TL	−0.002	0.049	0.973	−0.097	0.094	0.996	0.013	0.055	0.809	−0.094	0.120	0.982
	Time*TL	0.003	0.002	0.224	−0.002	0.007	0.468	0.004	0.002	0.106	−0.001	0.008	0.262
Temporal R	Time	−0.038	0.003	**<0.001** ^***^	−0.044	−0.032	**<0.001** ^***^	−0.038	0.003	**<0.001** ^***^	−0.043	−0.033	**<0.001** ^***^
	CE	−0.018	0.049	0.713	−0.114	0.078	0.901	−0.011	0.052	0.830	−0.112	0.090	0.982
	Time*CE	0.004	0.002	*0.056* ^†^	−0.000	0.009	0.145	0.005	0.002	**0.017** ^*^	0.001	0.009	**0.045** ^*^
	Time	−0.038	0.003	**<0.001** ^***^	−0.044	−0.032	**<0.001** ^***^	−0.037	0.003	**<0.001** ^***^	−0.043	−0.032	**<0.001** ^***^
	TL	−0.001	0.049	0.982	−0.097	0.095	0.996	−0.008	0.054	0.878	−0.114	0.097	0.982
	Time*TL	0.005	0.002	**0.048** ^*^	0.000	0.009	0.131	0.006	0.002	**0.008** ^**^	0.002	0.010	**0.022** ^*^
Cingulate L	Time	−0.028	0.002	**<0.001** ^***^	−0.032	−0.023	**<0.001** ^***^	−0.026	0.002	**<0.001** ^***^	−0.030	−0.022	**<0.001** ^***^
	CE	−0.027	0.055	0.618	−0.135	0.080	0.842	−0.022	0.058	0.699	−0.135	0.091	0.938
	Time*CE	−0.001	0.002	0.469	−0.005	0.002	0.739	−0.001	0.002	0.617	−0.004	0.002	0.911
	Time	−0.028	0.002	**<0.001** ^***^	−0.032	−0.024	**<0.001** ^***^	−0.026	0.002	**<0.001** ^***^	−0.030	−0.022	**<0.001** ^***^
	TL	0.027	0.055	0.623	−0.080	0.134	0.842	0.032	0.060	0.597	−0.086	0.150	0.911
	Time*TL	−0.001	0.002	0.664	−0.004	0.003	0.867	0.000	0.002	0.854	−0.003	0.004	0.982
Cingulate R	Time	−0.024	0.002	**<0.001** ^***^	−0.028	−0.020	**<0.001** ^***^	−0.023	0.002	**<0.001** ^***^	−0.027	−0.019	**<0.001** ^***^
	CE	−0.051	0.058	0.381	−0.164	0.063	0.641	−0.051	0.062	0.411	−0.173	0.071	0.704
	Time*CE	0.000	0.002	0.970	−0.003	0.003	0.996	0.001	0.002	0.656	−0.002	0.004	0.938
	Time	−0.024	0.002	**<0.001** ^***^	−0.028	−0.020	**<0.001** ^***^	−0.023	0.002	**<0.001** ^***^	−0.027	−0.019	**<0.001** ^***^
	TL	0.005	0.058	0.937	−0.109	0.118	0.996	0.010	0.065	0.876	−0.117	0.137	0.982
	Time*TL	0.001	0.002	0.576	−0.003	0.005	0.814	0.000	0.002	0.888	−0.003	0.004	0.982

hipp, hippocampus; Cx, cortex; L, left; R, right; CE, Closeness to Experience; TL, Tendence to Liabilities. Models are adjusted for age, sex, education, and APOE status of participants. Coefficients are expressed in standard deviation units (z-scores). Bold font indicates p-values <0.05. Italicized font indicates p-values <0.1 but larger than 0.05. ^†^*p* < 0.10; ^*^p < 0.05; ^**^p < 0.01; ^***^p < 0.001.

**Table 9:** Effects of time, personality factors and their interactions on white matter tract FA.

Dependent	Fixed factors	Full Sample						HC Sample					
		Coef.	Std. Err.	*p*-value	[0.025	0.975]	*p*_fdr	Coef.	Std. Err.	*p*-value	[0.025	0.975]	*p*_fdr
Fornix	Time	−0.036	0.004	**<0.001** ^***^	−0.045	−0.027	**<0.001** ^***^	−0.035	0.004	**<0.001** ^***^	−0.044	−0.027	**<0.001** ^***^
	CE	−0.006	0.059	0.914	−0.123	0.110	0.936	0.004	0.063	0.944	−0.118	0.127	0.970
	Time*CE	0.002	0.003	0.509	−0.005	0.009	0.815	0.002	0.003	0.640	−0.005	0.008	0.853
	Time	−0.035	0.004	**<0.001** ^***^	−0.044	−0.026	**<0.001** ^***^	−0.034	0.004	**<0.001** ^***^	−0.043	−0.026	**<0.001** ^***^
	TL	−0.026	0.059	0.660	−0.142	0.090	0.815	−0.030	0.062	0.634	−0.152	0.092	0.853
	Time*TL	−0.002	0.004	0.654	−0.009	0.005	0.815	−0.002	0.003	0.650	−0.008	0.005	0.853
Cingulate cingulum R	Time	−0.034	0.006	**<0.001** ^***^	−0.046	−0.023	**<0.001** ^***^	−0.031	0.006	**<0.001** ^***^	−0.043	−0.019	**<0.001** ^***^
	CE	−0.146	0.059	**0.014** ^*^	−0.263	−0.030	0.073	−0.179	0.061	**0.004** ^**^	−0.299	−0.058	**0.025** ^*^
	Time*CE	0.000	0.004	0.938	−0.008	0.009	0.938	0.004	0.005	0.374	−0.005	0.013	0.628
	Time	−0.035	0.006	**<0.001** ^***^	−0.046	−0.024	**<0.001** ^***^	−0.031	0.006	**<0.001** ^***^	−0.043	−0.020	**<0.001** ^***^
	TL	−0.047	0.060	0.438	−0.164	0.071	0.815	−0.090	0.062	0.149	−0.212	0.032	0.483
	Time*TL	0.002	0.005	0.591	−0.006	0.011	0.815	0.006	0.005	0.240	−0.004	0.015	0.561
Cingulate cingulum L	Time	−0.015	0.006	**0.020** ^*^	−0.027	−0.002	*0.083* ^†^	−0.009	0.006	0.138	−0.021	0.003	0.483
	CE	−0.183	0.060	**0.002** ^**^	−0.301	−0.065	**0.020** ^*^	−0.214	0.062	**0.001** ^**^	−0.335	−0.093	**0.004** ^**^
	Time*CE	−0.002	0.005	0.621	−0.012	0.007	0.815	−0.000	0.005	0.970	−0.009	0.009	0.970
	Time	−0.015	0.006	**0.018** ^*^	−0.027	−0.002	*0.083* ^†^	−0.009	0.006	0.134	−0.021	0.003	0.483
	TL	−0.041	0.061	0.506	−0.161	0.079	0.815	−0.091	0.063	0.150	−0.215	0.033	0.483
	Time*TL	−0.002	0.005	0.716	−0.012	0.008	0.859	0.000	0.005	0.933	−0.009	0.010	0.970
Cingulate Hippocampus R	Time	−0.007	0.008	0.382	−0.023	0.009	0.815	−0.011	0.009	0.186	−0.028	0.006	0.523
	CE	0.008	0.059	0.895	−0.108	0.123	0.936	0.014	0.061	0.821	−0.105	0.132	0.958
	Time*CE	−0.006	0.006	0.333	−0.018	0.006	0.815	−0.008	0.007	0.228	−0.021	0.005	0.561
	Time	−0.007	0.008	0.391	−0.023	0.009	0.815	−0.013	0.009	0.136	−0.030	0.004	0.483
	TL	−0.008	0.059	0.891	−0.124	0.108	0.936	−0.003	0.062	0.963	−0.125	0.119	0.970
	Time*TL	−0.006	0.007	0.374	−0.019	0.007	0.815	−0.007	0.007	0.292	−0.021	0.006	0.588
Cingulate Hippocampus L	Time	0.021	0.008	**0.012** ^*^	0.005	0.037	*0.072* ^†^	0.017	0.009	*0.061* ^†^	−0.001	0.034	0.319
	CE	−0.075	0.059	0.204	−0.191	0.041	0.713	−0.081	0.064	0.204	−0.206	0.044	0.535
	Time*CE	−0.003	0.007	0.622	−0.017	0.010	0.815	−0.005	0.007	0.485	−0.020	0.009	0.728
	Time	0.020	0.008	**0.009** ^**^	0.005	0.035	*0.064* ^†^	0.017	0.009	*0.052* ^†^	−0.000	0.034	0.312
	TL	0.020	0.063	0.747	−0.103	0.143	0.872	0.016	0.064	0.800	−0.108	0.141	0.958
	Time*TL	−0.005	0.006	0.450	−0.017	0.007	0.815	−0.008	0.007	0.268	−0.021	0.006	0.588
Uncinate R	Time	0.007	0.010	0.459	−0.012	0.026	0.815	0.010	0.010	0.322	−0.009	0.029	0.588
	CE	−0.062	0.062	0.318	−0.185	0.060	0.815	−0.042	0.065	0.522	−0.170	0.086	0.756
	Time*CE	−0.004	0.007	0.549	−0.019	0.010	0.815	−0.007	0.007	0.370	−0.021	0.008	0.628
	Time	0.007	0.010	0.455	−0.012	0.026	0.815	0.011	0.010	0.295	−0.009	0.030	0.588
	TL	−0.051	0.063	0.421	−0.174	0.073	0.815	−0.065	0.066	0.321	−0.193	0.063	0.588
	Time*TL	−0.004	0.007	0.560	−0.019	0.010	0.815	−0.006	0.008	0.474	−0.021	0.010	0.728
Uncinate L	Time	−0.005	0.010	0.577	−0.025	0.014	0.815	−0.002	0.011	0.818	−0.023	0.018	0.958
	CE	0.007	0.064	0.908	−0.117	0.132	0.936	−0.003	0.065	0.958	−0.132	0.125	0.970
	Time*CE	−0.007	0.008	0.340	−0.022	0.008	0.815	−0.007	0.008	0.402	−0.023	0.009	0.650
	Time	−0.005	0.010	0.614	−0.024	0.014	0.815	−0.002	0.011	0.866	−0.022	0.019	0.970
	TL	0.012	0.064	0.849	−0.113	0.138	0.936	0.019	0.066	0.773	−0.110	0.149	0.958
	Time*TL	−0.011	0.008	0.178	−0.026	0.005	0.679	−0.011	0.008	0.187	−0.028	0.005	0.523

L, left; R, right; CE, Closeness to Experience; TL, Tendence to Liabilities. Models are adjusted for age, sex, education, and *APOE* status of participants. Coefficients are expressed in standard deviation units (z-scores). Bold font indicates *p*-values <0.05. Italicized font indicates *p*-values <0.1 but larger than 0.05. ^†^*p* < 0.10; ^*^p < 0.05; ^**^p < 0.01; ^***^p < 0.001.

**Figure 4:**
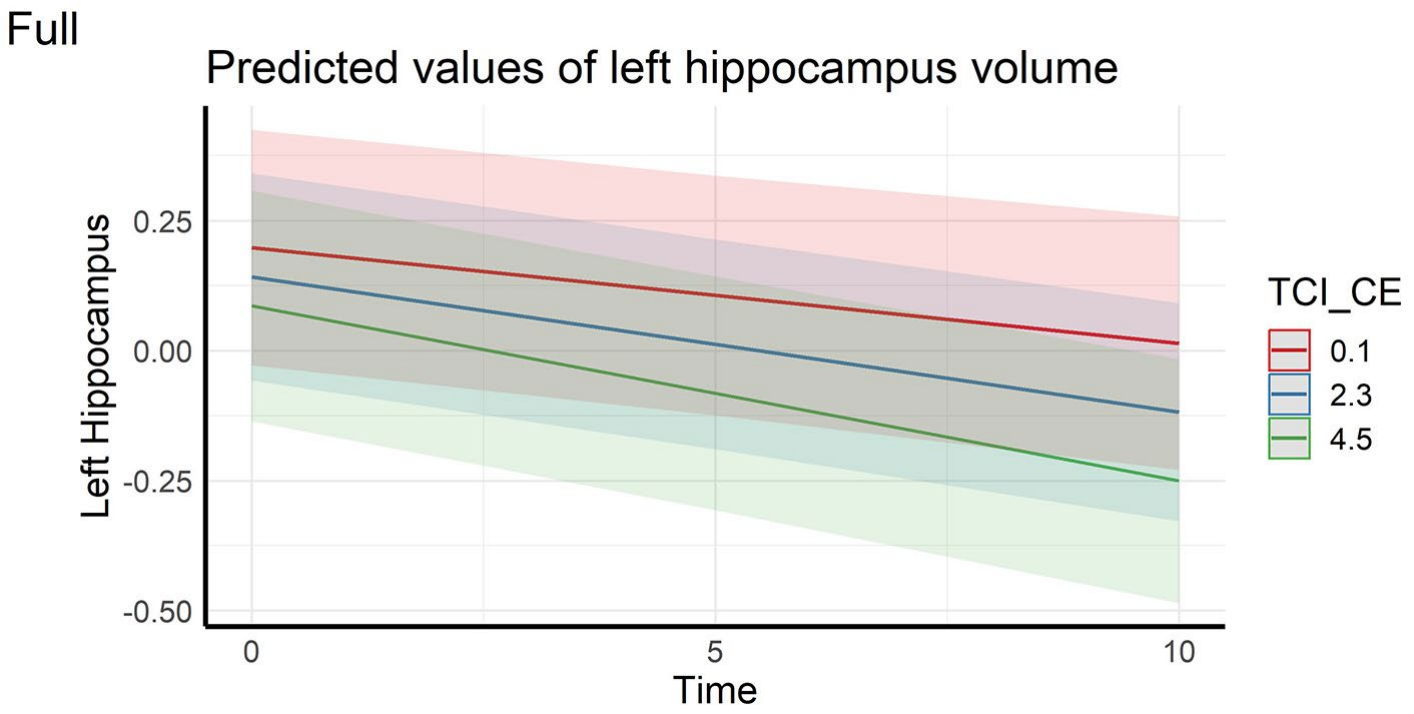
Interaction effect between Closeness to Experience and Time on left hippocampus volume in the full sample. TCI_CE, Closeness to Experience score assessed with Temperament and Character Inventory.

**Figure 5:**
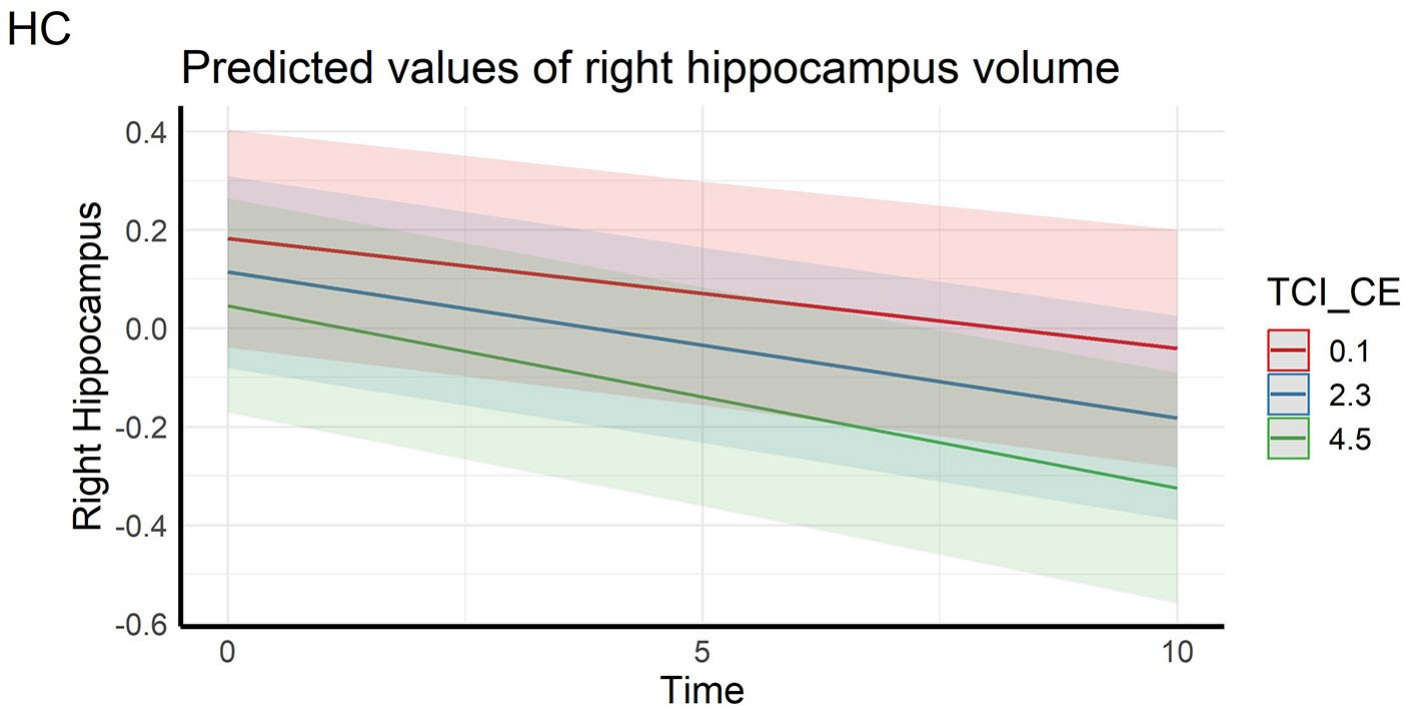
Interaction effect between Closeness to Experience and Time on right hippocampus volume in the healthy control subsample. HC, Healthy Control subsample; TCI_CE, Closeness to Experience score assessed with Temperament and Character Inventory.

Regarding the cortical and hippocampal volumes assessed with MRI, main effects of time on all structures (cortical and white matter volumes) were observed: volumes decrease over time (in full and HC samples). The left hippocampal volume decreased more over time with higher CE scores in full sample (Figure 4) whereas the right hippocampal volume decreased more over time with higher CE in HC (Figure 5). A nominally significant interaction between TL and time for right temporal volume did not survive FDR correction in the full sample, but in HC, the same structure unexpectedly decreased less over time as the CE and TL scores were higher (personality x time-interaction effects), even after FDR correction. No cross-sectional effects of personality factors on brain gray matter volumes were observed, neither were cross-sectional, nor longitudinal effects of personality factors observed for white matter volumes (see Supplementary Table S1).

Regarding analyses of white matter FA, main effects of time on fornix and right cingulate-cingulum FA were observed: FA decreased over time in both full and HC sample. Effects of time on left cingulate-cingulum and cingulate hippocampus FA in the full sample were nominally significant but did not survive FDR correction. Cross-sectional effects of CE on left cingulate-cingulum FA were significant in both groups; individuals with higher the CE had lower FA. In the full sample, the effect of CE on baseline right cingulate cingulum FA was nominally significant but did not survive the FDR correction, whereas it did so in the HC subsample. No longitudinal interaction effects with personality factors on FA were observed.

### 3.6 Control analysis for health conditions

Since much research has indicated that negative personality traits can have an adverse effect on health (Allen et al., 1993; Perkins et al., 1993; Brickman et al., 1996; Denollet et al., 2000; Erlen et al., 2011), we reran our models with the additional covariates of self-reported HBP, diabetes, and cardiovascular disease. Out of the significant effects reported above for the full sample, these adjustments rendered the interaction effects between time and the CE- (FDR-corrected value of *p* [*p*-FDR] = 0.099) and TL factors (*p*-FDR = 0.057) for verbal fluency performance non-significant. In the HC subsample, the main effects of the CE factor on vocabulary (*p*-FDR = 0.072) and verbal fluency (*p*-FDR = 0.057) performance, as well as the interaction effect between time and the CE factor on episodic memory (*p*-FDR = 0.057) did not survive consideration of health covariates. The other significant effects of personality factors on cognition, gray matter volume, and fractional anisotropy reported in the main analyses remained significant also after adjustment of health factors, and the corrected coefficients and *p*-values can be found in Supplementary Table S2. Thus, to summarize, most of the effects of personality factors on cognitive and brain aging remained significant after adjustment for common health conditions, albeit some of the effects on cognitive decline may have been partially mediated by somatic health.

### 3.7 Risk factors for AD dementia conversion

The results of the proportional hazards model are summarized in Table 10. Of the 1,325 participants included in the analysis, 142 developed a dementia state due to AD during their follow-up visits.

**Table 10:** Assessment of risk factors for dementia using Cox proportional hazards regression.

Variable	Hazard ratio	Lower 95%	Upper 95%	*p*-value	*p*-adjusted
Sex	0.40	0.27	0.59	<0.005	0.0075
Age	1.04	1.02	1.06	<0.005	0. 0075
Education	0.83	0.78	0.88	<0.005	0. 0075
*APOE*-ε4	3.41	2.43	4.77	<0.005	0. 0075
CE	1.02	0.94	1.10	0.72	0.72
TL	1.04	0.96	1.13	0.34	0.408

Exp(coef), exponential regression coefficient indicating increased (>1) or decreased (<1) risk for each additional point to the variable of interest; CE, Closeness to Experience; TL, Tendence to Liabilities.

The risk of developing AD dementia was significantly impacted by sex, where being male reduced the risk of developing a dementia state by 60%; age, where each additional year increased the risk of dementia by 4%; years of education, where each additional year of education reduced the risk by 17%; and *APOE* status, where being a carrier of an *APOE*-ε4 allele increased the risk by 3.41 times. Finally, neither of the personality factors (CE nor TL) significantly predicted the conversion to AD-type dementia in the Cox regression models adjusted for age, sex, education level and *APOE*-ε4 carriership.

## 4 Discussion

The current study examined the impact of personality factors predictive of mental disorders (Dell’Orco et al., 2018) on NPS and cognitive, as well as neuroanatomical trajectories of participants from a population-based longitudinal aging study. The results show that the revised personality factors CE and TL are associated with higher NPS, greater cognitive decline over time, as well as accelerated volume reduction of certain brain volumes such as the hippocampus.

Firstly, we demonstrate that two of the revised personality factors based on TCI, the CE and TL factors were associated cross-sectionally and longitudinally with depression and stress scores, and cross-sectionally with sleep disturbances. We thereby extend the initial observations made by Dell’Orco et al. (2018) in a younger clinical population with high psychiatric illness burden to an older population. Interestingly, in our study, associations were demonstrated when NPS and sleep disturbances were measured between 10 and 17 years after personality assessment with the TCI, thus demonstrating the predictive validity of the CE and TL personality factors. Personality is considered stable in healthy populations (Brändström et al., 2008), which was also the case for our revised personality factors (Table 5), whereas the NPS measured focus on manifestations that occurred over a limited period of time: the last year for the PSQ (Levenstein et al., 1993), the last 3 months for the KSQ (Kecklund and Åkerstedt, 1992), the last 2 weeks for the PHQ-9 (Kroenke et al., 2001) and the last week for the CES-D (Radloff, 1977). Thus, our findings further underscore the known association between personality traits and NPS (Archer et al., 2007; Rubio et al., 2013), and are in line with other work that has shown associations between negative personality traits like neuroticism with psychopathological symptoms such as depression and anxiety (Kotov et al., 2010; Andersen and Bienvenu, 2011; Jeronimus et al., 2016). It should be noted that in the Betula study, both personality and NPS are self-evaluated via questionnaires. Nevertheless, these data suggest an important relationship between these revised TCI personality factors and future NPS in a predominantly healthy older population.

Regarding cognition, we hypothesized that participants with higher personality scores on factors associated with higher NPS scores, CE and TL, would have poorer and more rapidly declining performance on age-sensitive cognitive domains such as memory, executive (verbal fluency) and visuoconstructive performance, whereas vocabulary ability was expected more stable across aging. Our results were reported separately for the full sample, including converters to AD-dementia, and a healthy subsample of non-converters, in order to elucidate the effects of neurodegenerative disease on the associations. In the full sample, greater scores on CE and TL were associated with greater decline in episodic memory, vocabulary and verbal fluency. The fact that vocabulary, a crystallized ability, showed similar effects as the fluid cognitive domains is in line with recent findings on the strong dependency between aging-related declines in fluid and crystallized abilities (Tucker-Drob et al., 2022). In the healthy control subset, however, only the longitudinal effect of CE on episodic memory was significant. This suggests that these personality factors have a greater negative impact on cognitive decline when considering participants converting to AD. The fact that the effect of CE and TL were attenuated in the healthy subset compared to the full sample could indicate that the effects may have been partially driven by individuals with AD-related cognitive decline. However, our follow-up Cox regression analysis did not indicate that CE or TL at baseline were significant predictors of AD conversion. The loss of significance in the healthy subset may have been caused by loss of statistical power due to the smaller sample size, but it is also possible that cognitive decline is a more sensitive measure of emerging neurocognitive dysfunction than conversion to dementia. Nevertheless, the CE personality factor had a significant effect on 25-year memory decline regardless of AD conversion status, showing that declines in some cognitive domains can be reliably predicted in healthy older adults. However, influence of subclinical AD-pathology on such declines cannot be conclusively ruled out, as polygenic risk scores for AD risk have previously been shown to predict cognitive decline in carefully screened healthy participants from this study cohort (Kauppi et al., 2020). Furthermore, it is noteworthy that the studied personality factors did not show significant associations with decline in all age-sensitive cognitive domains, such as Block Design, which in fact showed the steepest longitudinal decline in our sample. Finally, significant cross-sectional effects of TL and/or CE were observed for all cognitive domains except memory in the full sample, and for vocabulary and verbal fluency in the HC sample. These effects are in line with previous studies showing associations between negative personality traits and NPS, and lower cognitive abilities (Archer et al., 2007; Dotson et al., 2010; Saczynski et al., 2010; Low et al., 2013; Peters et al., 2013; Rubio et al., 2013; Terracciano et al., 2014; Forrester et al., 2016; Acosta et al., 2018; Liew, 2019; Burhanullah et al., 2020).

As reviewed in the introduction, most previous work on the association between personality traits and age-related cognitive declines have been based on the Big Five model. In their conception and definition, CE and TL factors seem close to Neuroticism or the opposite of Extraversion. CE is related to anxiety, tendency to pessimism, low novelty seeking, whereas TL is characterized by inability to change, low autonomy, low self-esteem. As a reminder, the neuroticism trait of the Big Five theory, i.e., tendency to experience negative and stressful life events, is positively correlated with the harm avoidance trait assessed by the TCI (tendency toward an inhibitory response to signals of aversive stimuli that lead to avoidance of punishment and non-reward) (Capanna et al., 2012). Moreover, CE and TL factors share with harm avoidance the characteristic of being a risk factor for mental disorders (Dell’Orco et al., 2018). It has been shown that high neuroticism is associated with increased risk of mild cognitive impairment in individuals, as well as accelerated memory and executive function decline (Crowe et al., 2006; Low et al., 2013; Terracciano et al., 2014; Caselli et al., 2016, but also see Wettstein et al., 2019). Thus, our findings for CE, and to a lesser extent TL, support some of these trends in their longitudinal effects on normative cognitive aging and tentatively also for the risk of cognitive impairment because of their greater effects sizes in the sample including AD converting participants. This supports the hypothesis that negative personality traits are associated with lower or declining memory and executive performance in aging. An avenue for future studies could be to further explore also positive personality traits, other than agreeableness and openness, for their potential protective effects on neurocognitive aging outcomes as observed in prior studies (Terracciano et al., 2014).

With regards to brain characteristics, in both full and HC samples, we observed expected aging-related declines in cortical gray matter, white matter and hippocampal volumes over time. Negative longitudinal effects were observed for CE on left hippocampal volume in the full sample and on right hippocampal volume in the HC sample, so that higher CE scores were related to faster decline. The results for the hippocampi are in line with the cognitive results, showing a more rapid decline in episodic memory in individuals with higher CE scores, when considering the well-established role of the hippocampus for episodic memory processing (Eichenbaum, 2017). Previous studies have shown mixed and predominantly null findings regarding the association between negative personality characteristics such as neuroticism and hippocampus volume in healthy young adults (Gray et al., 2018; Liu et al., 2021), and in older adults (Jackson et al., 2011). However, there is robust evidence from animal and human studies that psychopathological disorders such as depression and posttraumatic stress are linked to reduced hippocampal volumes (Wise et al., 2017). In animal models for instance, long term stress has been shown to cause dendritic atrophy, synaptic loss, and reduced neurogenesis in the hippocampus (Radley and Morrison, 2005). In human cross-sectional studies, the direction of causality has been difficult to infer, as small hippocampal volumes have also been shown to be a vulnerability factor for stress-related disorders rather than a consequence of such disorders (Gilbertson et al., 2002; Lindgren et al., 2016). Our present data, on the contrary, are consistent with a model in which negative personality traits predispose to higher burden of NPS, in turn exacerbating hippocampal atrophy and memory decline during aging. Importantly, since personality was assessed 10–17 years prior to NPS assessment, and 10–15 prior to the baseline neuroimaging session, our data demonstrate strong temporal precedence of personality characteristics over neurocognitive outcomes.

Other work has associated personality traits in healthy older individuals with prefrontal, orbitofrontal, cingular, insular and temporal cortical volumes and amygdala volumes, both cross-sectionally and longitudinally (Kaasinen et al., 2005; Jackson et al., 2011; Kapogiannis et al., 2013; Liu et al., 2013; Giannakopoulos et al., 2020a,b, 2022), but our findings did not indicate any associations between these regions and CE or TL. We did however observe a negative cross-sectional effect of the CE factor on the cingulate-cingulum FA in both the full and HC subsamples, and the right cingulate-cingulum in the HC only, so that higher CE was associated with reduced white matter integrity as indicted by FA. The association of the cingulate with different personality traits such as neuroticism has been previously demonstrated in young and middle age adulthood with cross-sectional analyses (Xu and Potenza, 2012; Bauer et al., 2016; Prillwitz et al., 2018; Sanjari Moghaddam et al., 2020). This could suggest that this personality-brain association is an indication of a lifetime stable trait, as opposed to arising as a consequence of aging. Other data showed associations between personality traits and other types of white matter degeneration in participants with MCI: white matter lesions were greater in individuals with low conscientiousness or high neuroticism (Duron et al., 2014). However, in contradiction to our results these data did not find associations with hippocampal atrophy, suggesting that personality changes in individuals at risk of developing AD would be associated with white matter lesions rather than medial temporal regions, which could induce dysexecutive disorders (Duron et al., 2014). In addition to structural personality associations, other data suggest a relationship with stages of neurofibrillary aggregation, particularly more advanced stages in participants with lower agreeableness and higher neuroticism (Terracciano et al., 2013).

With regards to the mechanistic links between personality and the aging brain, there are several points to consider. Personality traits have been cross-sectionally associated with several brain networks in adulthood, notably involving the prefrontal and cingulate regions (Xu and Potenza, 2012; Bauer et al., 2016; Prillwitz et al., 2018; Sanjari Moghaddam et al., 2020). Certain acquired brain lesions or neurodegenerative diseases of these regions lead to personality changes and disturbances, as in the case of head trauma or frontotemporal degeneration (Salloway et al., 2008; Rascovsky et al., 2011). However, in our study personality was measured 10 to 15 years prior to the first neuroimaging assessment, making it less probable, albeit not impossible, that neurodegenerative processes influenced the personality assessments. Alternatively, personality could also be linked to brain aging through health conditions. Several studies have shown associations between high neuroticism and an increased risk of chronic diseases such as heart disease (arterial stenosis, myocardial infarction, coronary heart disease), HIV, diabetes, hypertension, etc., (Allen et al., 1993; Perkins et al., 1993; Brickman et al., 1996; Denollet et al., 2000; Erlen et al., 2011), all of which are conditions that also negatively influence brain health. The fact that some of the associations between personality and cognitive function were attenuated by addition of health covariates (HBP, cardiovascular disease, and diabetes) in our data supports the idea that the effects of personality traits on brain function may be partially mediated through somatic health conditions. However, most of the personality associations to cognition and brain characteristics remained significant after control of health covariates, which suggest that there are likely several mechanisms contributing these associations, including health conditions not accounted for in our analyses. Thus, several factors may contribute to the relationship between personality and age-related brain and cognitive declines, and somatic health conditions is likely one.

Despite the longitudinal associations between CE and TL and cognitive decline, our Cox proportional hazard analyses did not show any indications of the CE and TL personality factors as risk factors of AD-type dementia. Instead, in line with much previous research the risk increased with age, being a woman, and carrying *APOE*-ε4, while years of education was a protective factor. In contrast, other studies have shown that certain personality traits are risk factors for dementia or cognitive decline, in particular neuroticism (Terracciano et al., 2014; Caselli et al., 2016; Sapkota et al., 2016). Also, mid-life personality was demonstrated as dementia risk factor by Johansson et al. (2014); after 38 years of follow-up in women without dementia higher neuroticism was associated with a higher risk of dementia and distress (Johansson et al., 2014). However, after control for the distress factor, the association between neuroticism and the risk of dementia decreased, suggesting that the risk is higher when neuroticism is associated with distress. These data suggest that neuroticism may be a reliable factor in predicting the risk of conversion to dementia, whereas CE and TL factors seem more reliable for predicting longitudinal aging-related cognitive decline which may be caused by multiple other factors other than dementia-related neurodegenerative processes, including hippocampal atrophy related to neuropsychiatric disorders as discussed above. This point is further underscored by the fact that the association between CE and memory decline were also demonstrated in the HC subsample which should have a lower prevalence of AD-related neuropathology, but in which the memory association was attenuated by addition of health covariates.

### 4.1 Strengths and limitations

This study was based on relatively large and population-based sample and included comprehensive measurement of constructs, for example a variety of cognitive abilities, and long-term follow up. A particular noteworthy feature is that personality was assessed 10–17 years before NPS, and 10–15 years prior to neuroimaging outcomes. One potential caveat, however, is that at the time of the personality assessment, the presence or absence of NPS was not available. Thus, it is not possible to conclusively determine whether personality assessments were influenced by NPS, or whether personality factors were driving later development of NPS. Another limitation is that AD diagnoses were not informed by amyloid beta or tau biomarkers. Nevertheless, the comprehensive diagnostic evaluation considered medical records as well as extensive cognitive and health assessments, resulting in a reliable clinical characterization (Nyberg et al., 2020). Lack of objective biomarkers in the full sample also prevented clear distinction between normative aging-related cognitive decline, and cognitive decline caused by subclinical AD- or other dementia-related neuropathologies. However, such distinctions are precarious to make since many pathologies associated with dementia are common also in cognitively healthy elderly who never develop dementia (Driscoll and Troncoso, 2011). For this reason, a continuous view of cognitive aging and dementia is increasingly being endorsed, where the difference is one of degree rather than kind (Tucker-Drob, 2019). Finally, a further limitation of the current study was that the neuroimaging subsample was significantly smaller than the original sample and had very few participants converting to AD. Nevertheless, significant effects converging on the medial temporal lobe memory system were still observed in the neuroimaging subsample.

## 5 Conclusion

This work is the first to consider the revised psychiatric disorders-related factors of the TCI as potential predictors of NPS in aging and age-related cognitive and cerebral decline. The results showed that *Closeness to Experience* (CE: avoidance of new stimuli, high anxiety, pessimistic anticipation, low reward seeking) and *Tendence to Liabilities* factors (TL: inability to change, low autonomy, unaware of the value of their existence) were associated with higher levels of depressive symptoms, stress (CE), sleep disturbance (TL), and greater decline in memory, vocabulary and verbal fluency. Moreover, higher CE was also associated with greater memory decline and faster right hippocampal volume reduction in the subsample not converting to dementia. Thus, the results indicate that these personality traits related to psychiatric symptoms are also predictors of NPS in aging as well as accelerated age-related cognitive decline, even in the absence of neurodegenerative disease. Some associations between personality and cognition were attenuated by adjusting for cardiovascular and hypertension indicators, suggesting that these effects may be partly mediated by somatic health. This work has deepened the clinical knowledge of the revised TCI as a self-assessment instrument for NPS assessment in aging. In addition, the current work adds to the mounting evidence of the clinical value of personality assessments for prediction of neurocognitive declines in healthy and pathological aging. Taken together, our findings further emphasize the importance of personality in neurocognitive aging and underscore the need for an integrative (biopsychosocial) perspective of normal and pathological age-related cognitive decline.

## Data availability statement

The data analyzed in this study is subject to the following licenses/restrictions: the Betula study dataset employed in the present study is available from the corresponding author or the study manager upon reasonable request, provided that the data transfer is in agreement with the European Union legislation on the General Data Protection Regulation and Umeå University data protection policies. Requests to access these datasets should be directed to https://www.umu.se/en/research/projects/betula---aging-memory-and-dementia/collaboration-on-betula-data/.

## Ethics statement

The studies involving humans were approved by the local ethics board at Umeå University. The studies were conducted in accordance with the local legislation and institutional requirements. The participants provided their written informed consent to participate in this study.

## Author contributions

LR: Conceptualization, Formal analysis, Methodology, Writing – original draft, Data curation. MR: Writing – review & editing. RA: Writing – review & editing. AH: Supervision, Writing – review & editing. SP: Conceptualization, Supervision, Visualization, Writing – review & editing.

## Footnotes

^1^ http://www.fmrib.ox.ac.uk/fsl
