# Supplementary material for: Revised Temperament and Character Inventory factors predict neuropsychiatric symptoms and aging-related cognitive decline across 25 years
Source: Front Aging Neurosci. 2024 Feb 21;16:1335336. doi: 10.3389/fnagi.2024.1335336 (PMC10915205; doi:10.3389/fnagi.2024.1335336)
Supplement: Supplementary file 1 [file Data_Sheet_1.docx]

Supplementary Table 1. Effects of time, personality factors and their interactions on white matter volumes

|  |  | **Full Sample** | | | | | | **HC Sample** | | | | | |
| --- | --- | --- | --- | --- | --- | --- | --- | --- | --- | --- | --- | --- | --- |
| Dependent | Fixed factors | Coef. | Std.Err. | *p*-value | [0.025 | 0.975] | p_fdr | Coef. | Std.Err. | *p*-value | [0.025 | 0.975] | p_fdr |
| Frontal WM L | Time | -0.037 | 0.002 | **<.001***** | -0.041 | -0.033 | **<.001***** | -0.033 | 0.002 | **<.001***** | -0.036 | -0.029 | **<.001***** |
|  | CE | -0.039 | 0.052 | 0.454 | -0.140 | 0.063 | 0.726 | -0.026 | 0.051 | 0.610 | -0.126 | 0.074 | 0.911 |
|  | Time*CE | -0.000 | 0.002 | 0.821 | -0.004 | 0.003 | 0.973 | -0.001 | 0.001 | 0.553 | -0.004 | 0.002 | 0.884 |
|  | Time | -0.037 | 0.002 | **<.001***** | -0.041 | -0.033 | **<.001***** | -0.033 | 0.002 | **<.001***** | -0.037 | -0.029 | **<.001***** |
|  | TL | -0.064 | 0.051 | 0.217 | -0.164 | 0.037 | 0.462 | -0.052 | 0.053 | 0.331 | -0.156 | 0.053 | 0.611 |
|  | Time*TL | 0.000 | 0.002 | 0.901 | -0.003 | 0.004 | 0.995 | 0.000 | 0.002 | 0.917 | -0.003 | 0.003 | 0.986 |
| Frontal WM R | Time | -0.039 | 0.002 | **<.001***** | -0.044 | -0.035 | **<.001***** | -0.036 | 0.002 | **<.001***** | -0.040 | -0.032 | **<.001***** |
|  | CE | -0.021 | 0.049 | 0.667 | -0.118 | 0.076 | 0.866 | -0.019 | 0.050 | 0.703 | -0.117 | 0.079 | 0.938 |
|  | Time*CE | -0.003 | 0.002 | 0.124 | -0.006 | 0.001 | 0.290 | -0.002 | 0.002 | 0.183 | -0.005 | 0.001 | 0.383 |
|  | Time | -0.039 | 0.002 | **<.001***** | -0.044 | -0.035 | **<.001***** | -0.036 | 0.002 | **<.001***** | -0.040 | -0.032 | **<.001***** |
|  | TL | -0.053 | 0.049 | 0.282 | -0.149 | 0.043 | 0.531 | -0.047 | 0.052 | 0.364 | -0.150 | 0.055 | 0.647 |
|  | Time*TL | -0.003 | 0.002 | *0.063†* | -0.007 | 0.000 | 0.159 | -0.003 | 0.002 | 0.101 | -0.006 | 0.001 | 0.254 |
| Temporal WM L | Time | -0.038 | 0.003 | **<.001***** | -0.044 | -0.032 | **<.001***** | -0.033 | 0.003 | **<.001***** | -0.038 | -0.028 | **<.001***** |
|  | CE | -0.022 | 0.050 | 0.665 | -0.119 | 0.076 | 0.866 | -0.025 | 0.048 | 0.599 | -0.121 | 0.070 | 0.911 |
|  | Time*CE | -0.004 | 0.002 | *0.089†* | -0.009 | 0.001 | 0.220 | -0.003 | 0.002 | 0.138 | -0.007 | 0.001 | 0.317 |
|  | Time | -0.038 | 0.003 | **<.001***** | -0.044 | -0.032 | **<.001***** | -0.033 | 0.003 | **<.001***** | -0.038 | -0.028 | **<.001***** |
|  | TL | -0.029 | 0.050 | 0.560 | -0.126 | 0.068 | 0.814 | -0.035 | 0.051 | 0.484 | -0.134 | 0.064 | 0.802 |
|  | Time*TL | -0.004 | 0.002 | 0.144 | -0.009 | 0.001 | 0.329 | -0.002 | 0.002 | 0.258 | -0.007 | 0.002 | 0.515 |
| Temporal WM R | Time | -0.037 | 0.002 | **<.001***** | -0.041 | -0.033 | **<.001***** | -0.036 | 0.002 | **<.001***** | -0.039 | -0.032 | **<.001***** |
|  | CE | -0.033 | 0.051 | 0.519 | -0.134 | 0.067 | 0.792 | -0.016 | 0.054 | 0.768 | -0.123 | 0.091 | 0.978 |
|  | Time*CE | 0.000 | 0.002 | 0.985 | -0.003 | 0.003 | 0.996 | -0.000 | 0.002 | 0.896 | -0.003 | 0.003 | 0.982 |
|  | Time | -0.037 | 0.002 | **<.001***** | -0.041 | -0.033 | **<.001***** | -0.036 | 0.002 | **<.001***** | -0.039 | -0.032 | **<.001***** |
|  | TL | -0.066 | 0.051 | 0.198 | -0.165 | 0.034 | 0.432 | -0.055 | 0.057 | 0.329 | -0.166 | 0.056 | 0.611 |
|  | Time*TL | 0.000 | 0.002 | 0.999 | -0.003 | 0.003 | 0.999 | 0.000 | 0.002 | 0.775 | -0.003 | 0.004 | 0.978 |
| Cingulate WM L | Time | -0.043 | 0.002 | **<.001***** | -0.048 | -0.039 | **<.001***** | -0.042 | 0.002 | **<.001***** | -0.046 | -0.038 | **<.001***** |
|  | CE | -0.039 | 0.049 | 0.429 | -0.136 | 0.058 | 0.698 | -0.028 | 0.053 | 0.592 | -0.131 | 0.075 | 0.911 |
|  | Time*CE | -0.000 | 0.002 | 0.939 | -0.004 | 0.003 | 0.996 | -0.001 | 0.002 | 0.734 | -0.004 | 0.003 | 0.952 |
|  | Time | -0.043 | 0.002 | **<.001***** | -0.048 | -0.039 | **<.001***** | -0.042 | 0.002 | **<.001***** | -0.046 | -0.038 | **<.001***** |
|  | TL | -0.095 | 0.049 | *0.052†* | -0.190 | 0.001 | 0.138 | -0.100 | 0.054 | *0.066†* | -0.207 | 0.007 | 0.171 |
|  | Time*TL | -0.001 | 0.002 | 0.572 | -0.005 | 0.003 | 0.814 | -0.000 | 0.002 | 0.900 | -0.004 | 0.003 | 0.982 |
| Cingulate WM R | Time | -0.037 | 0.003 | **<.001***** | -0.044 | -0.031 | **<.001***** | -0.036 | 0.003 | **<.001***** | -0.041 | -0.030 | **<.001***** |
|  | CE | -0.059 | 0.053 | 0.267 | -0.163 | 0.045 | 0.526 | -0.050 | 0.057 | 0.374 | -0.161 | 0.061 | 0.652 |
|  | Time*CE | 0.000 | 0.003 | 0.881 | -0.005 | 0.006 | 0.995 | -0.000 | 0.002 | 0.996 | -0.004 | 0.004 | 0.996 |
|  | Time | -0.037 | 0.003 | **<.001***** | -0.044 | -0.031 | **<.001***** | -0.036 | 0.003 | **<.001***** | -0.041 | -0.030 | **<.001***** |
|  | TL | -0.044 | 0.053 | 0.403 | -0.148 | 0.060 | 0.667 | -0.042 | 0.059 | 0.477 | -0.158 | 0.074 | 0.802 |
|  | Time*TL | 0.001 | 0.003 | 0.600 | -0.004 | 0.007 | 0.835 | -0.001 | 0.002 | 0.794 | -0.005 | 0.004 | 0.982 |

Legend: L = Left; R = Right; WM = White matter; CE = Closeness to Experience; TL = Tendence to Liability. Models are adjusted for age, sex, education, and *APOE* status of participants. †p<.10; *p<.05; ***p<.001

Supplementary Table 2. Effects of personality factors and their interactions on cognition, grey matter, and fractional anisotropy considering health covariates.

|  |  | **Full Sample** | | | | | | **HC Sample** | | | | | |
| --- | --- | --- | --- | --- | --- | --- | --- | --- | --- | --- | --- | --- | --- |
| Dependent | Fixed Factor | Coef. | Std.Err. | *p*-value | [0.025 | 0.975] | p_fdr | Coef. | Std.Err. | *p*-value | [0.025 | 0.975] | p_fdr |
| **Cognitive** | | | | | | | | | | | | | |
| Mem | CE | -0.052 | 0.024 | **0.027*** | -0.099 | -0.006 | **0.046*** | -0.054 | 0.025 | **0.029*** | -0.103 | -0.006 | *0.072†* |
|  | Time*CE | -0.005 | 0.002 | **0.002**** | -0.008 | -0.002 | **0.008**** | -0.004 | 0.002 | **0.017*** | -0.007 | -0.001 | *0.057†* |
|  | TL | -0.021 | 0.024 | 0.378 | -0.067 | 0.025 | 0.5 | -0.043 | 0.025 | *0.085†* | -0.091 | 0.006 | 0.169 |
|  | Time*TL | -0.005 | 0.002 | **0.004**** | -0.008 | -0.001 | **0.017*** | -0.002 | 0.002 | 0.175 | -0.005 | 0.001 | 0.280 |
| Voc | CE | -0.061 | 0.024 | **0.012*** | -0.109 | -0.013 | **0.029*** | -0.056 | 0.026 | **0.028*** | -0.107 | -0.006 | *0.072†* |
|  | Time*CE | -0.002 | 0.001 | **0.024*** | -0.005 | -0.000 | **0.045*** | -0.002 | 0.001 | 0.104 | -0.004 | 0.000 | 0.189 |
|  | TL | -0.062 | 0.024 | **0.011*** | -0.109 | -0.014 | **0.029*** | -0.074 | 0.026 | **0.004**** | -0.124 | -0.024 | **0.019*** |
|  | Time*TL | -0.003 | 0.001 | **0.016*** | -0.005 | -0.000 | **0.032*** | -0.002 | 0.001 | 0.110 | -0.004 | 0.000 | 0.189 |
| Block | CE | -0.069 | 0.025 | **0.005**** | -0.118 | -0.021 | **0.017*** | -0.058 | 0.027 | **0.030*** | -0.110 | -0.006 | *0.072†* |
|  | Time*CE | -0.001 | 0.001 | 0.438 | -0.004 | 0.002 | 0.500 | -0.001 | 0.001 | 0.415 | -0.004 | 0.002 | 0.474 |
|  | TL | -0.059 | 0.025 | **0.016*** | -0.108 | -0.011 | **0.032*** | -0.049 | 0.027 | *0.065†* | -0.101 | 0.003 | 0.143 |
|  | Time*TL | -0.001 | 0.001 | 0.292 | -0.004 | 0.001 | 0.412 | -0.001 | 0.001 | 0.362 | -0.004 | 0.001 | 0.434 |
| Flu | CE | -0.057 | 0.021 | **0.006**** | -0.098 | -0.017 | **0.017*** | -0.053 | 0.022 | **0.015*** | -0.097 | -0.010 | *0.057†* |
|  | Time*CE | -0.002 | 0.001 | *0.066†* | -0.005 | 0.000 | 0.099 | -0.002 | 0.001 | 0.223 | -0.004 | 0.001 | 0.334 |
|  | TL | -0.014 | 0.021 | 0.486 | -0.055 | 0.026 | 0.531 | -0.015 | 0.022 | 0.498 | -0.058 | 0.028 | 0.543 |
|  | Time*TL | -0.003 | 0.001 | **0.035*** | -0.005 | -0.000 | *0.057†* | -0.001 | 0.001 | 0.281 | -0.004 | 0.001 | 0.376 |
| **Grey Matter Volumes** | | | | | | | | | | | | | |
| Hipp L | CE | -0.053 | 0.052 | 0.312 | -0.156 | 0.050 | 0.565 | -0.050 | 0.055 | 0.361 | -0.159 | 0.058 | 0.659 |
|  | Time*CE | -0.007 | 0.003 | **0.006**** | -0.012 | -0.002 | **0.017*** | -0.006 | 0.003 | **0.028*** | -0.011 | -0.001 | *0.074†* |
|  | TL | -0.029 | 0.052 | 0.578 | -0.132 | 0.074 | 0.828 | -0.004 | 0.058 | 0.942 | -0.118 | 0.109 | 0.999 |
|  | Time*TL | -0.001 | 0.003 | 0.621 | -0.007 | 0.004 | 0.850 | -0.002 | 0.003 | 0.471 | -0.008 | 0.004 | 0.787 |
| Hipp R | CE | -0.078 | 0.053 | 0.141 | -0.182 | 0.026 | 0.338 | -0.058 | 0.052 | 0.258 | -0.159 | 0.043 | 0.528 |
|  | Time*CE | -0.006 | 0.003 | **0.041*** | -0.012 | -0.000 | 0.112 | -0.007 | 0.003 | **0.011*** | -0.013 | -0.002 | **0.032*** |
|  | TL | 0.003 | 0.054 | 0.950 | -0.102 | 0.108 | 1.000 | 0.014 | 0.054 | 0.798 | -0.092 | 0.120 | 0.935 |
|  | Time*TL | -0.003 | 0.003 | 0.343 | -0.010 | 0.003 | 0.600 | -0.003 | 0.003 | 0.290 | -0.009 | 0.003 | 0.557 |
| Temporal R | CE | -0.016 | 0.050 | 0.742 | -0.114 | 0.081 | 0.937 | -0.009 | 0.052 | 0.866 | -0.110 | 0.093 | 0.990 |
|  | Time*CE | 0.004 | 0.002 | *0.055†* | -0.000 | 0.009 | 0.142 | 0.005 | 0.002 | **0.023** | 0.001 | 0.009 | *0.064†* |
|  | TL | -0.000 | 0.049 | 1.000 | -0.097 | 0.097 | 1.000 | -0.006 | 0.054 | 0.913 | -0.112 | 0.100 | 0.999 |
|  | Time*TL | 0.005 | 0.002 | **0.029*** | 0.001 | 0.010 | *0.083†* | 0.006 | 0.002 | **0.006**** | 0.002 | 0.011 | **0.016*** |
| **Fractional Anisotropy** | | | | | | | | | | | | | |
| Cingulate cingulum L | CE | -0.157 | 0.059 | **0.008**** | -0.273 | -0.040 | *0.087†* | -0.187 | 0.062 | **0.002**** | -0.307 | -0.066 | **0.026*** |
|  | Time*CE | 0.002 | 0.004 | 0.648 | -0.007 | 0.011 | 0.916 | 0.005 | 0.005 | 0.256 | -0.004 | 0.015 | 0.784 |
|  | TL | -0.061 | 0.060 | 0.307 | -0.179 | 0.056 | 0.877 | -0.102 | 0.063 | 0.104 | -0.225 | 0.021 | 0.511 |
|  | Time*TL | 0.006 | 0.005 | 0.206 | -0.003 | 0.015 | 0.877 | 0.009 | 0.005 | *0.088†* | -0.001 | 0.018 | 0.511 |
| Cingulate cingulum R | CE | -0.192 | 0.061 | **0.002**** | -0.311 | -0.073 | **0.022*** | -0.221 | 0.062 | **<0.001***** | -0.343 | -0.100 | **0.005**** |
|  | Time*CE | -0.001 | 0.005 | 0.813 | -0.011 | 0.008 | 0.916 | 0.001 | 0.005 | 0.854 | -0.009 | 0.010 | 0.963 |
|  | TL | -0.053 | 0.062 | 0.387 | -0.174 | 0.067 | 0.877 | -0.102 | 0.064 | 0.109 | -0.226 | 0.023 | 0.511 |
|  | Time*TL | 0.002 | 0.005 | 0.729 | -0.008 | 0.012 | 0.916 | 0.003 | 0.005 | 0.504 | -0.007 | 0.013 | 0.847 |

Legend: Mem = Episodic Memory; Voc = Vocabulary; Bloc = Bloc Design; Flu = Verbal Fluency; CE = Closeness to Experience; TL = Tendence to Liabilities, Hipp = hippocampus; L = Left; R = Right. Models are adjusted for age, sex, education, cardiovascular disease, high blood pressure, diabetes and *APOE* status of participants. †p<.10; *p<.05; **p<.01; ***p<.001
